# Evaluation of the design of the influenza-like illness sentinel surveillance system in Brazil

**DOI:** 10.1590/0102-311XEN028823

**Published:** 2024-07-29

**Authors:** Laís Picinini Freitas, Cláudia Torres Codeço, Leonardo Soares Bastos, Daniel Antunes Maciel Villela, Oswaldo Gonçalves Cruz, Antonio Guilherme Pacheco, Flavio Codeço Coelho, Raquel Martins Lana, Luiz Max Fagundes de Carvalho, Roberta Pereira Niquini, Walquiria Aparecida Ferreira de Almeida, Daiana Araújo da Silva, Felipe Cotrim de Carvalho, Marcelo Ferreira da Costa Gomes

**Affiliations:** 1 Programa de Computação Científica, Fundação Oswaldo Cruz, Rio de Janeiro, Brasil.; 2 Escola de Matemática Aplicada, Fundação Getulio Vargas, Rio de Janeiro, Brasil.; 3 Barcelona Supercomputing Center, Barcelona, España.; 4 Instituto Federal de Educação, Ciência e Tecnologia do Rio de Janeiro, Rio de Janeiro, Brasil.; 5 Secretaria de Vigilância em Saúde, Ministério da Saúde, Brasília, Brasil.

**Keywords:** Severe Acute Respiratory Syndrome, Human Influenza, Sentinel Surveillance, Síndrome Respiratorio Agudo Grave, Gripe Humana, Vigilancia de Guardia

## Abstract

The influenza-like illness (ILI) sentinel surveillance operates in Brazil to identify respiratory viruses of public health relevance circulating in the country and was first implemented in 2000. Recently, the COVID-19 pandemic reinforced the importance of early detection of the circulation of new viruses in Brazil. Therefore, an analysis of the design of the ILI sentinel surveillance is timely. To this end, we simulated a sentinel surveillance network, identifying the municipalities that would be part of the network according to the criteria defined in the design of the ILI sentinel surveillance and, based on data from tested cases of severe acute respiratory illness (SARI) from 2014 to 2019, we drew samples for each sentinel municipality per epidemiological week. The draw was performed 1,000 times, obtaining the median and 95% quantile interval (95%QI) of virus positivity by Federative Unit and epidemiological week. According to the ILI sentinel surveillance design criteria, sentinel units would be in 64 municipalities, distributed mainly in capitals and their metropolitan areas, recommending 690 weekly samples. The design showed good sensitivity (91.65% considering the 95%QI) for qualitatively detecting respiratory viruses, even those with low circulation. However, there was important uncertainty in the quantitative estimate of positivity, reaching at least 20% in 11.34% of estimates. The results presented here aim to assist in evaluating and updating the ILI sentinel surveillance design. Strategies to reduce uncertainty in positivity estimates need to be evaluated, as does the need for greater spatial coverage.

## Resumen

La vigilancia centinela de la enfermedad tipo infuenza (ETI) funciona en Brasil para identificar los virus respiratorios de importancia para la salud pública que circulan en el país y comenzó a ser implementada en 2000. Recientemente, la pandemia de COVID-19 ha reforzado la importancia de la detección temprana de la circulación de nuevos virus en el territorio brasileño. Así, se hace oportuno un análisis del diseño de la vigilancia centinela de la ETI. Para ello, simulamos una red centinela identificando los municipios que formarían parte de la red según los criterios definidos en el diseño de la vigilancia centinela de la ETI y, a partir de los datos de casos testados de infección respiratoria aguda grave (IRAG) de 2014 a 2019, se extrajeron muestras para cada municipio centinela por semana epidemiológica. El sorteo se repitió 1.000 veces y se obtuvo la mediana y el intervalo cuantílico del 95% (IC95%) de la positividad por virus, por Unidad Federativa y semana epidemiológica. Según los criterios del diseño de la vigilancia centinela de la ETI, unidades centinelas estarían en 64 municipios, distribuidas principalmente en capitales y zonas metropolitanas de las capitales, preconizando 690 muestras semanales. El diseño presentó una buena sensibilidad (total de 91,65% considerando el IC95%) para la detección cualitativa de los virus respiratorios, incluso los de baja circulación. Sin embargo, hubo una importante incertidumbre en la estimación cuantitativa de la positividad, alcanzando al menos el 20% en el 11,34% de las estimaciones. Los resultados presentados aquí tienen como objetivo ayudar en la evaluación y actualización del diseño de la red centinela. Es necesario evaluar las estrategias para reducir la incertidumbre en las estimaciones de positividad, al igual que la necesidad de una mayor cobertura espacial.

## Introduction

The influenza-like illness (ILI) sentinel surveillance began to be implemented in Brazil in 2000 to identify respiratory viruses of public health relevance circulating in the country, guide the composition of the seasonal influenza vaccine, and generate alerts for unusual events, such as the emergence of a new virus ^1^ ^,^ ^2^ ^,^ ^3^. It integrates the national surveillance of influenza and other respiratory viruses, including the universal surveillance of hospitalized cases and deaths due to severe acute respiratory illness (SARI), implemented in 2009. Data from both surveillances are recorded in an official information system called Influenza Epidemiological Surveillance System (SIVEP-Gripe, acronym in Portuguese), which is part of the Brazilian Health Informatics Department (DATASUS, acronym in Portuguese) of the Brazilian Ministry of Health.

At the Brazilian Ministry of Health, ILI sentinel surveillance system is formed by a network of healthcare units, following the guidelines of the World Health Organization (WHO) ^1^ ^,^ ^4^. There are other forms of sentinel surveillance, such as in countries where surveillance is formed by healthcare professionals ^5^. According to data from the European Centre for Disease Prevention and Control (ECDC), since 2015, all member states have reported data for seasonal influenza surveillance from ILI cases in primary care ^5^. Several of these countries use not only sentinel units but also voluntary reporting data from nonsentinel units for status monitoring and viral identification, a common scenario within countries that form the European region of the WHO ^6^. In the United States, the Centers for Disease Control and Prevention (CDC) also uses ILI sentinel surveillance to monitor cases of respiratory infection requiring outpatient care ^7^. The CDC highlights that only a subset of cases collect a sample for viral identification, with the main focus of sentinel surveillance being monitoring the trend and volume of cases of general respiratory infections, not being virus-specific ^7^. In Europe, a sampling strategy is also adopted to collect samples for testing, but not all ILI cases reported in the sentinel network are tested ^8^. In the Americas, of the 38 countries and territories evaluated by 2021 by the Pan American Health Organization (PAHO), 25 had surveillance for ILI and 31 for SARI, with the vast majority employing sentinel surveillance and laboratory testing of only a subset of cases of ILI ^9^.

To strengthen sentinel surveillance in Brazil after the A(H1N1)pdm09 influenza pandemic, *Ordinance n. 183* ^10^ of the Brazilian Ministry of Health was published, dated January 30, 2014, which determines, in Chapter 5, Art. 28, §1, the criteria for the distribution of healthcare units that form the network of the ILI sentinel surveillance. These units are mandatorily healthcare services that must include urgency and emergency units and serve people of all age groups. Until 2019, sentinel surveillance defined a case of ILI as an individual with fever, even if self-reported, followed by cough or sore throat and with the onset of symptoms in the last seven days, treated at a sentinel healthcare unit. Regarding laboratory analysis, five samples of nasopharyngeal secretion from ILI cases were recommended per week in each sentinel unit ^2^ ^,^ ^10^. These samples are sent to public laboratories to be tested against a panel of respiratory viruses that have changed over the years, including new viruses. With this sampling, the data captured are expected to be representative of the Federative Unit.

Unlike ILI surveillance, which is based on sampling, all cases and deaths that meet the definition of SARI must be reported to SIVEP-Gripe and tested for a panel of respiratory viruses, regardless of the healthcare unit. SARI cases until 2019 were defined as cases of hospitalized or deceased individuals, regardless of previous hospitalization, with the same symptoms as ILI plus dyspnea or O_2_ saturation lower than 95% or respiratory distress ^3^. The respiratory viruses found in SARI surveillance do not necessarily represent the circulating viral population since some viruses lead to milder clinical conditions that SARI surveillance would not readily identify. Thus, ILI sentinel surveillance (mild cases) and universal surveillance for SARI (severe cases) are complementary as they cover a broad spectrum of respiratory syndromes.

The COVID-19 pandemic, a disease caused by the SARS-CoV-2, further reinforced the importance of monitoring respiratory syndromes for early detection of the circulation of new viruses in Brazil. The emerging virus spread quickly across the country, reaching regions far from large urban centers within a few weeks ^11^. Due to changes in human circulation patterns and an increased risk of introducing and spreading new viruses and variants, it is opportune to analyze the currently proposed sentinel surveillance network to identify strengths and weaknesses that can support new designs. Therefore, the main objective of this work is to evaluate the performance of the design of the ILI sentinel surveillance regarding its ability to detect the prevalence of respiratory viruses by Federative Unit.

## Methodology

### Data

In Brazil, the available data source with the best coverage of information on the circulation of respiratory viruses comes from universal SARI surveillance. This surveillance is capable of monitoring Brazil’s seasonality of ILI ^12^ and has become essential to monitor the expansion of SARS-CoV-2 ^13^ ^,^ ^14^ ^,^ ^15^ and assist in planning national immunization against COVID-19 ^16^. In the absence of an unbiased data source, this study assumed that the distribution of SARI cases by viral subtype captured by universal SARI surveillance is representative of the actual spatial distribution of respiratory viruses in Brazil. Thus, a sentinel network with adequate spatial distribution is expected to detect this viral distribution per Federative Unit, which is the spatial resolution for which the ILI sentinel surveillance network was designed to be representative ^2^ ^,^ ^3^ ^,^ ^17^ ^,^ ^18^. To test this hypothesis, we (1) identified which municipalities would be eligible to compose the sentinel network; (2) calculated how many sentinel units and how many weekly samples would be recommended in each eligible municipality; and (3) simulated the data collection process carried out by the network sentinel in a scenario in which the viral population per week and Federative Unit comes from a sample with replacement of the viral composition of SARI cases on the same date and location. To do so, we took as a basis *Ordinance n. 183* ^10^ of the Brazilian Ministry of Health, Chapter 5, Art. 28, §1, and other documents from the Ministry of Health that complement the information in Ordinance ^2^ ^,^ ^10^ ^,^ ^18^.

Data on SARI cases registered in SIVEP-Gripe were obtained from InfoGripe (http://infogripe.fiocruz.br/), an initiative to monitor and present alert levels for SARI cases ^19^. The project is the result of a partnership between researchers from the Scientific Computing Program, Oswaldo Cruz Foundation (PROCC/FIOCRUZ, acronym in Portuguese), the School of Applied Mathematics, Getulio Vargas Foundation (EMap/FGV, acronym in Portuguese), and the Health Surveillance Secretariat, Brazilian Ministry of Health.

Data on SARI cases from 2014 to 2019 were used, according to the year of onset of symptoms, totaling 214,162 records. Only laboratory-tested cases were selected from the total, resulting in 178,106 cases (83.2%). During the studied period, the available laboratory tests covered the following viruses: adenovirus, influenza A, influenza B, parainfluenza 1, parainfluenza 2, parainfluenza 3, parainfluenza 4, respiratory syncytial virus, metapneumovirus, rhinovirus, and bocavirus. The positivity of each virus among the tested SARI cases from 2014 to 2019 is shown at the Supplementary Material (Figure S1 https://cadernos.ensp.fiocruz.br/static//arquivo/csp-0288-23-sup-een028823_5054.pdf).

Population estimates for 2019 from the Brazilian Ministry of Health, made available by DATASUS (https://datasus.saude.gov.br/), were also used.

### Analyses

#### Identification of municipalities eligible to form the sentinel network

Initially, the municipalities that would be eligible to be part of the ILI sentinel surveillance network ^10^ were identified, namely: (1) all capitals of the Federative Unit; (2) municipalities with a population greater than 300,000 inhabitants in the South Region; and (3) municipalities with more than 300,000 inhabitants in the metropolitan areas of the capitals of the other regions. Then, the number of sentinel units and weekly samples recommended for each municipality was calculated ^2^ ^,^ ^10^: five weekly samples for each sentinel unit, with (1) one unit for every 500,000 inhabitants in the capitals and (2) one unit in other municipalities in the network. The number of weekly samples expected per 1 million inhabitants per Federative Unit was also calculated.

#### Simulated sentinel surveillance

The set of 178,106 laboratory-tested SARI cases reported in SIVEP-Gripe was stratified by week and municipality of notification (2014-2019). From this set, *n*_*m,t*_ cases were drawn in each sentinel municipality *m* and epidemiological week *t*, following the aforementioned criteria of the sentinel surveillance strategy. The draw assumed that all cases reported in the sentinel municipality have the same probability of being captured by the sentinel units present there. On the other hand, cases reported in municipalities without sentinel units have zero probability of being captured by the sentinel network. The drawing process, with replacement, was performed a 1,000 times to obtain measures of uncertainty. We call the resulting dataset simulated sentinel surveillance.

From the total number of SARI cases captured by the simulated sentinel, *P*_*v,t,i,k*_ was calculated, defined as the positivity of virus *v* in week *t*, for each Federative Unit *i* and repetition *k* (Equation 1). As the process occurred 1,000 times, with *k* = 1, 2, ..., 1,000, there are 1,000 values describing the distribution of positivity for each virus by Federative Unit and week. From *P*_*v,t,i,k*_ , the median and 95% quantile interval (95%QI) of positivity for each virus *v* per week *t* and Federative Unit *i* were calculated.

$${Ρ}_{v,t,i,k}=\frac{\sum_{m\in i}{positive}_{m,v,t,k}}{\sum_{m\in i}n_{m,t}}\times 100$$

The absolute error *E*_*v,t,i,k*_ (Equation 2) was calculated to analyze the quality of the indicator generated by the simulated sentinel, comparing each positivity value *P*_*v,t,i,k*_ of the simulated sentinel with the “true” positivity (*ϕ*_*v,i,t*_ ), calculated from the total SARI data present in the universe of reported and laboratory-tested cases.

$$E_{v,t,i,k}=\left|P_{v,t,i,k}-\phi_{v,i,t}\right|$$

From *E*_*v,t,i,k*_ , the median absolute error was calculated for each virus *v* per week *t* and Federative Unit *i*.

Maps were created to compare errors in positivity estimates between Federative Unit and between periods of the year with greater or lesser respiratory virus activity. Using the Moving Epidemic Method (MEM) ^20^ ^,^ ^21^ implemented in InfoGripe ^22^, periods of each year in each Federative Unit were classified as epidemic (weeks of higher activity) or interepidemic (weeks of lower activity). The ECDC routinely uses this method of classifying influenza activity ^20^. The average of absolute errors was used for each virus per period, calculated as the sum of absolute errors divided by the number of weeks to compare the performance of the sentinel in periods of high and low activity.

As there are more than ten respiratory viruses tested in the laboratory by the surveillance system, we classified the viruses into two groups to facilitate outcomes interpretation: those with greater and lesser circulation, selecting one from each group for presentation. For this classification, we considered a 2% cutoff point for positivity in the SARI data universe (Supplementary Material - Figure S1 https://cadernos.ensp.fiocruz.br/static//arquivo/csp-0288-23-sup-een028823_5054.pdf). The two viruses selected to represent the groups with the high and low circulation, respectively, were influenza A (positivity = 16.4%) and parainfluenza 3 (positivity = 1.2%). The results for the other viruses are available in the Supplementary Material (Figures S2-S11 https://cadernos.ensp.fiocruz.br/static//arquivo/csp-0288-23-sup-een028823_5054.pdf).

We used R version 4.0.4 (http://www.r-project.org) and the *tidyverse* package ^23^ to organize and analyze the data. The graphs and maps were created in R using the *ggplot2* package ^24^.

### Ethical aspects

This study used nonidentifiable data that can be found unrestricted and publicly on the OpenDATASUS page (https://opendatasus.saude.gov.br/).

## Results

According to the design of the ILI sentinel surveillance, the strategy would include 138 units in 64 municipalities, targetting 690 samples per week. Of these 64 municipalities, 10 would be concentrated only in the metropolitan area of São Paulo. On average, sentinel municipalities should have two sentinel units, ranging from 1 to 25. The list of municipalities with the target number of units and samples, according to the design, can be found in Supplementary Material (Table S1 https://cadernos.ensp.fiocruz.br/static//arquivo/csp-0288-23-sup-een028823_5054.pdf).

**Figure 1:**
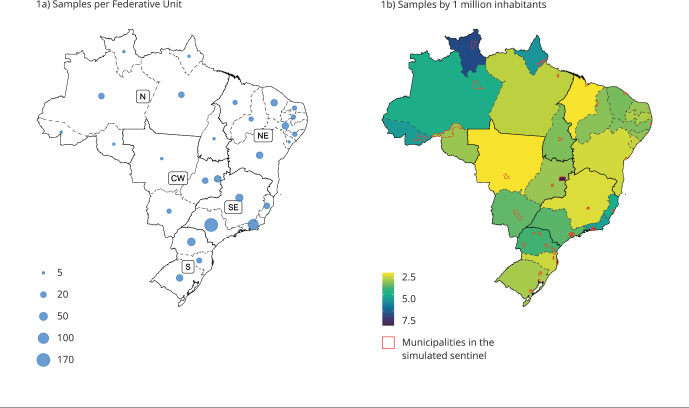
Weekly samples recommended by the influenza-like illness sentinel surveillance design, by Federative Unit, and per 1 million inhabitants, Brazil.

CW: Central-West; N: North; NE: Northeast; S: South; SE:Southeast.

This design predicts more weekly samples collected in the Federative Unit of the South and Southeast regions, where most of the Brazilian population is concentrated (Figure 1a). The proposed sampling corresponds to 3.3 per million inhabitants, ranging 1.4-9.9 per Federative Unit (Figure 1b). The Federative Unit with the lowest number of samples per population would be Maranhão (1.4 samples per million inhabitants), Mato Grosso (1.4), and Minas Gerais (1.8). In contrast, those with the highest number would be the Federal District (9.9), Roraima (8.2), and Amapá (5.9). Figure 1b also highlights the municipalities that meet the criteria to join the sentinel network as designed. In most Federative Units, these municipalities correspond to the capitals or municipalities neighboring the capitals. Only Paraná would have sentinel municipalities more widely distributed throughout the state.

**Figure 2:**
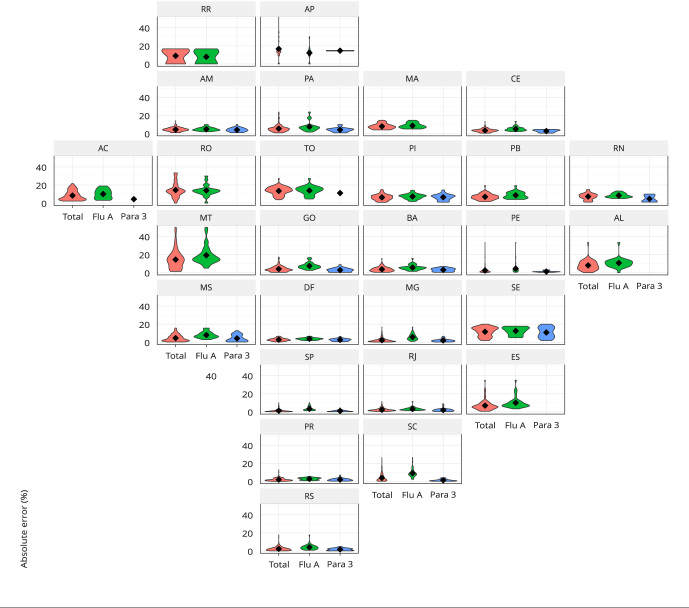
Distribution of absolute errors in viral positivity detected by simulated sentinel surveillance concerning the actual values obtained from the severe acute respiratory illness (SARI) surveillance system for all viruses (Total), for influenza A (Flu A), and parainfluenza 3 (Para 3), by epidemiological week and Federative Unit, Brazil, 2014-2019.

Figure 2 shows the distribution of absolute errors in the positivity rate of simulated sentinel surveillance by Federative Unit and week for all viruses, for influenza A and parainfluenza 3. The simulated sentinel in the Federal Units of the South and Southeast (except Espírito Santo) and the Federal District are noted to present minor absolute errors, with the distribution of errors more concentrated at values close to zero. Only in Amapá did the upper limit of the distribution of absolute errors exceed 50%. The errors in Mato Grosso and Roraima presented a wider distribution range.

Federative Units: AC: Acre; AL: Alagoas; AM: Amazonas; AP: Amapá; BA: Bahia; CE: Ceará; DF: Federal District; ES: Espírito Santo; GO: Goiás; MA: Maranhão; MG: Minas Gerais; MS: Mato Grosso do Sul; MT: Mato Grosso; PA: Pará; PB: Paraíba; PE: Pernambuco; PI: Piauí; PR: Paraná; RJ: Rio de Janeiro; RN: Rio Grande do Norte; RO: Rondônia; RR: Roraima; RS: Rio Grande do Sul; SC: Santa Catarina; SE: Sergipe; SP: São Paulo; TO: Tocantins.

**Figure 3:**
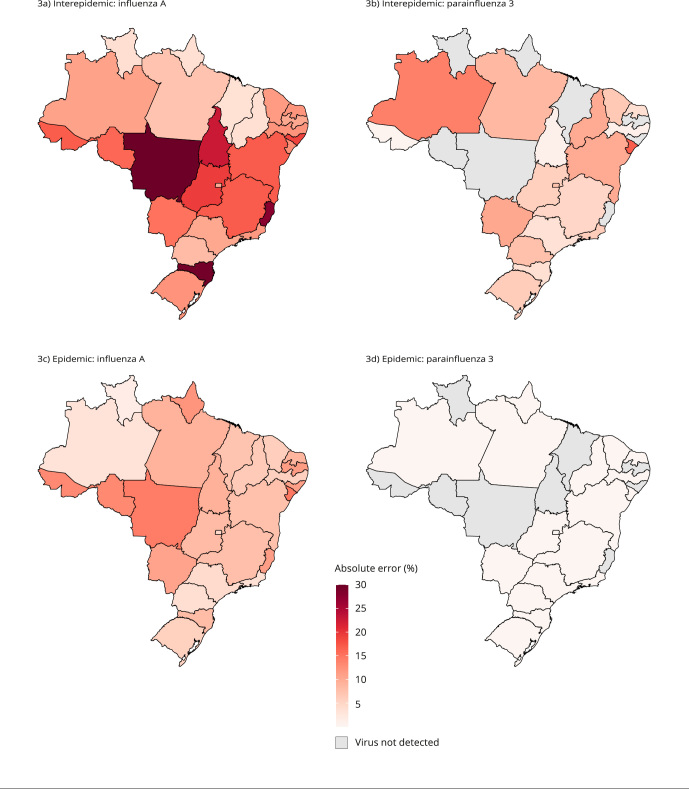
Absolute errors in the simulated influenza-like illness sentinel surveillance concerning the total severe acute respiratory illness (SARI) data for influenza A and parainfluenza 3, by epidemiological period and Federative Unit, Brazil, 2014-2019.

Figure 3 shows that, in general, the absolute errors were greater in the interepidemic period and for the influenza A virus (with higher circulation), compared with parainfluenza 3 (with lower circulation). For influenza A, absolute errors ranged from 3.6% (Maranhão) to 29.9% (Mato Grosso) in the interepidemic period and from 1.7% (Roraima) to 14.7% (Sergipe) in the epidemic period. For parainfluenza 3, absolute errors ranged from 0.4% (Acre) to 16.9% (Sergipe) in the interepidemic period and from 0.1% (Minas Gerais) to 2.9% (Amapá) in the epidemic period. The parainfluenza 3 virus was not detected in the total SARI data in eight Federative Units in the interepidemic period and nine in the epidemic period (gray areas in Figures 3b and 3d).

**Figure 4:**
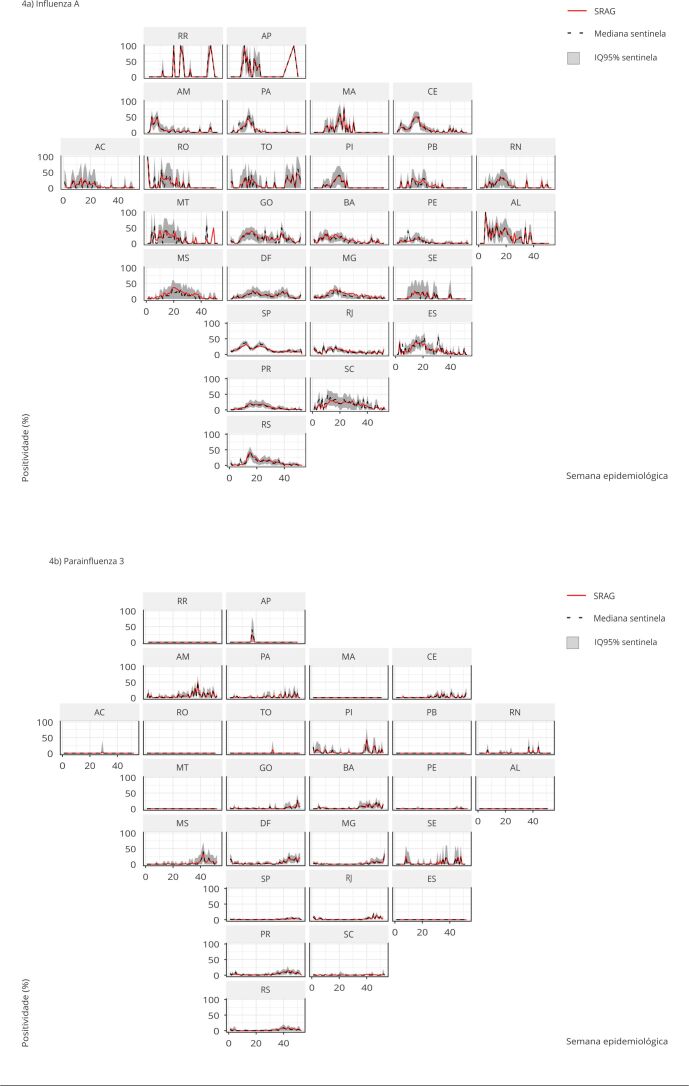
Positivity of influenza a and parainfluenza 3 obtained from the simulated influenza-like illness sentinel surveillance (median and 95% quantile interval - 95%QI) compared with the reference positivity obtained from the total of reported cases of severe acute respiratory illness (SARI) by Federative Unit and epidemiological week, Brazil, 2014-2019.

The presence of respiratory viruses (i.e., positivity greater than zero) was correctly detected by simulated sentinel surveillance in 91.65% of the total observations, considering the 95%QI. Considering only the median sentinel surveillance positivity, this value drops to 57.97%. Generally, the actual positivity values for influenza A and parainfluenza 3 were within the 95%QI range of the simulated sentinel surveillance positivity (Figure 4). For the influenza A virus, Rio de Janeiro, São Paulo, Paraná, and Rio Grande do Sul presented lower uncertainties, while in states such as Amapá, Rondônia, Mato Grosso, and Sergipe, the 95%QI range was greater than 50% in some weeks (Figure 4a). In seven Federative Units (Roraima, Maranhão, Rondônia, Paraíba, Mato Grosso, Alagoas, and Espírito Santo), the parainfluenza 3 virus was not detected in the SARI data universe in any week. Despite the low positivity, the simulated sentinel surveillance detected the presence of parainfluenza 3 when it was circulating (Figure 4b).

Federative Units: AC: Acre; AL: Alagoas; AM: Amazonas; AP: Amapá; BA: Bahia; CE: Ceará; DF: Federal District; ES: Espírito Santo; GO: Goiás; MA: Maranhão; MG: Minas Gerais; MS: Mato Grosso do Sul; MT: Mato Grosso; PA: Pará; PB: Paraíba; PE: Pernambuco; PI: Piauí; PR: Paraná; RJ: Rio de Janeiro; RN: Rio Grande do Norte; RO: Rondônia; RR: Roraima; RS: Rio Grande do Sul; SC: Santa Catarina; SE: Sergipe; SP: São Paulo; TO: Tocantins.

## Discussion

In this study, we sought to evaluate whether the ILI sentinel surveillance, considering its current design ^2^ ^,^ ^10^, would be capable of identifying the prevalence of different respiratory viruses by Federative Unit and week in Brazil. We considered SARI surveillance data, which has coverage throughout the country, to be representative of the “real” prevalence of respiratory viruses. Based on these data, we simulated data captured by sentinel surveillance as proposed in the abovementioned ordinance. Overall, we found that the simulated sentinel surveillance could qualitatively detect the presence of respiratory viruses in the Federative Units, but the positivity estimates were not accurate.

The evaluation presented here considers a perfect implementation of the sentinel network as designed ^2^ ^,^ ^10^. Therefore, it is not an assessment of the ILI sentinel surveillance network as it actually operates. In practice, there are challenges in maintaining active sentinel units and sending the recommended five weekly samples, as well as problems arising from operationalization such as quality of the nasopharyngeal sample collected, selection of ILI cases, storage and transportation of samples, access to laboratories, and quality of recorded data, among others ^25^ ^,^ ^26^. Furthermore, we consider 100% fulfillment of the target of five weekly samples, while a minimum of 80% is required to transfer funds. This implies that the results presented here correspond to an upper limit of the performance of this system.

Based on simulations carried out, the capacity of the sentinel network as designed for temporal and spatial monitoring of the composition of the viral population at the Federative Unit level was verified, i.e., with the detection of the circulating viral types. The good sensitivity presented for the parainfluenza 3 virus indicates that sentinel surveillance is adequate to detect less prevalent or intermittently occurring viruses. This result is essential to fulfill the objective of characterizing viruses for vaccine composition purposes, for example.

We generally observed lower absolute errors in Federative Units in the South and Southeast regions and the Federal District (Figure 2). This probably reflects the representativeness of the sentinel network sampling since more weekly samples are recommended in these locations (Figure 1a), and/or there is a high number of samples per 1 million inhabitants (Figure 1b). Regarding the estimate of positivity by the simulated sentinel, there is a large uncertainty for most viruses in most Federative Units (Figure 4). Its important to remember, the estimates calculated here stem from an ideal application of the current sentinel design without losing samples or units. Still, the uncertainty of positivity estimates was high in many weeks for some states. Furthermore, assessing the estimates’ precision is impossible when the positivity in the SARI data is zero. Overall, these results suggest that the current desing of the sentinel network is inadequate for the quantitative characterization of prevalence. A possible explanation for this result arises from the bias caused by the noninclusion of other municipalities in the sentinel network. For example, 86.5% of SARI cases are in municipalities not covered by the sentinel network.

According to SIVEP-Gripe, in 2017, there were 115 sentinel units in Brazil (ranging from one to seven units per municipality), which is 16.6% less than would be expected according to the design ^2^ ^,^ ^10^. Furthermore, these units were distributed in 67 municipalities, of which only 42 (64.6%) would be selected if these criteria were met. In the current design, some states concentrate sentinel units (São Paulo, Rio de Janeiro, Paraná, and Rio Grande do Sul), while the country has large uncovered spaces. According to the design of the ILI sentinel surveillance, only the South Region there is a plan for the establishment of sentinel units within the states ^2^ ^,^ ^10^. In other regions, only the capitals and some municipalities in metropolitan areas are covered. Even in the Southern states, it is clear that only in Paraná would there be eligible municipalities with greater territorial dispersion covering the state’s east, west, and north regions. In Santa Catarina and Rio Grande do Sul, the eligible municipalities outside the metropolitan area of the capitals are concentrated on the coast, in addition to the mountainous region in Rio Grande do Sul. The entire central and western region of these two states is uncovered. Among the country’s 118 health macroregions, 80 (67.8%) would not have any representation in the sentinel network according to the current design.

When revising the current protocol for distributing sentinel units in Brazil, we suggest using simulations to compare different protocols and evaluate their cost-effectiveness and efficacy. Proposals in the literature use mobility networks to identify strategic points ^27^. Another development path is the use of weighting to correct positivity estimates ^28^ ^,^ ^29^. Alternative models of sentinel networks that combine population representation with more uniform geographic coverage can also be explored ^30^.

The spatial and temporal dynamics of respiratory viruses are complex and variable, strongly influenced by climate, population characteristics, and population mobility patterns ^12^ ^,^ ^31^. Furthermore, global patterns of viral emergence and circulation also strongly determine national epidemiological dynamics. The emergence of COVID-19 showed the importance of sentinel networks for long-term monitoring of the virological characterization of SARS-CoV-2, as occurs with influenza.
