# Supplementary material for: Evaluation of the design of the influenza-like illness sentinel surveillance system in Brazil
Source: Cad Saude Publica. 2024 Jul 29;40(6):e00028823. doi: 10.1590/0102-311XEN028823 (PMC11321611; doi:10.1590/0102-311XEN028823)
Supplement: Supplementary file 1 [file 1678-4464-csp-40-06-EN028823-s.pdf]

Table S1 Number and name of municipalities that would be included in the influenza-like illness sentinel surveillance network by recommended number of sentinel units in the municipality and of weekly samples for collection, according to *Ordinance n. 183* of the Brazilian Ministry of Health.

Recommended number of sentinel units in the municipality	Recommended number of weekly samples	Number of eligible municipalities	Name of municipalities eligible to be part of the sentinel network
1	5	46	Rio Branco/AC, Macapá/AP, Caucaia/CE, Cariacica/ES, Serra/ES, Vila Velha/ES, Vitória/ES, Aparecida De Goiânia/GO, Betim/MG, Contagem/MG, Ribeirão Das Neves/MG, Cuiabá/MT, Ananindeua/PA, Jaboatão Dos Guararapes/PE, Olinda/PE, Paulista/PE, Cascavel/PR, Londrina/PR, Maringá/PR, Ponta Grossa/PR, São José Dos Pinhais/PR, Belford Roxo/RJ, Duque De Caxias/RJ, Niterói/RJ, Nova Iguacu/RJ, São Gonçalo/RJ, São João De Meriti/RJ, Porto Velho/RO, Boa Vista/RR, Canoas/RS, Caxias Do Sul/RS, Pelotas/RS, Blumenau/SC, Florianópolis/SC, Joinville/SC, Aracaju/SE, Carapicuíba/SP, Diadema/SP, Guarulhos/SP, Itaquaquecetuba/SP, Mauá/SP, Mogi Das Cruzes/SP, Osasco/SP, Santo André/SP, São Bernardo Do Campo/SP, Palmas/TO
2	10	6	Maceió/AL, São Luís/MA, Campo Grande/MS, João Pessoa/PB, Teresina/PI, Natal/RN
3	15	4	Goiânia/GO, Belém/PA, Recife/PE, Porto Alegre/RS
4	20	2	Manaus/AM, Curitiba/PR
5	25	2	Fortaleza/CE, Belo Horizonte/MG
6	30	2	Salvador/BA, Brasília/DF
13	65	1	Rio De Janeiro/RJ
25	125	1	São Paulo/SP

Federative Units: AC: Acre State; AL: Alagoas State; AM: Amazonas State; AP: Amapá State; BA: Bahia State; CE: Ceará State; DF: Distrito Federal; ES: Espírito Santo State; GO: Goiás State; MA: Maranhão State; MG: Minas Gerais State; MS: Mato Grosso do Sul State; MT: Mato Grosso State; PA: Pará State; PB: Paraíba State; PE: Pernambuco State; PI: Piauí State; PR: Paraná State; RJ: Rio de Janeiro State; RN: Rio Grande do Norte State; RO: Rondônia State; RR: Roraima State; RS: Rio Grande do Sul State; SC: Santa Catarina State; SE: Sergipe State; SP: São Paulo State; TO: Tocantins State.

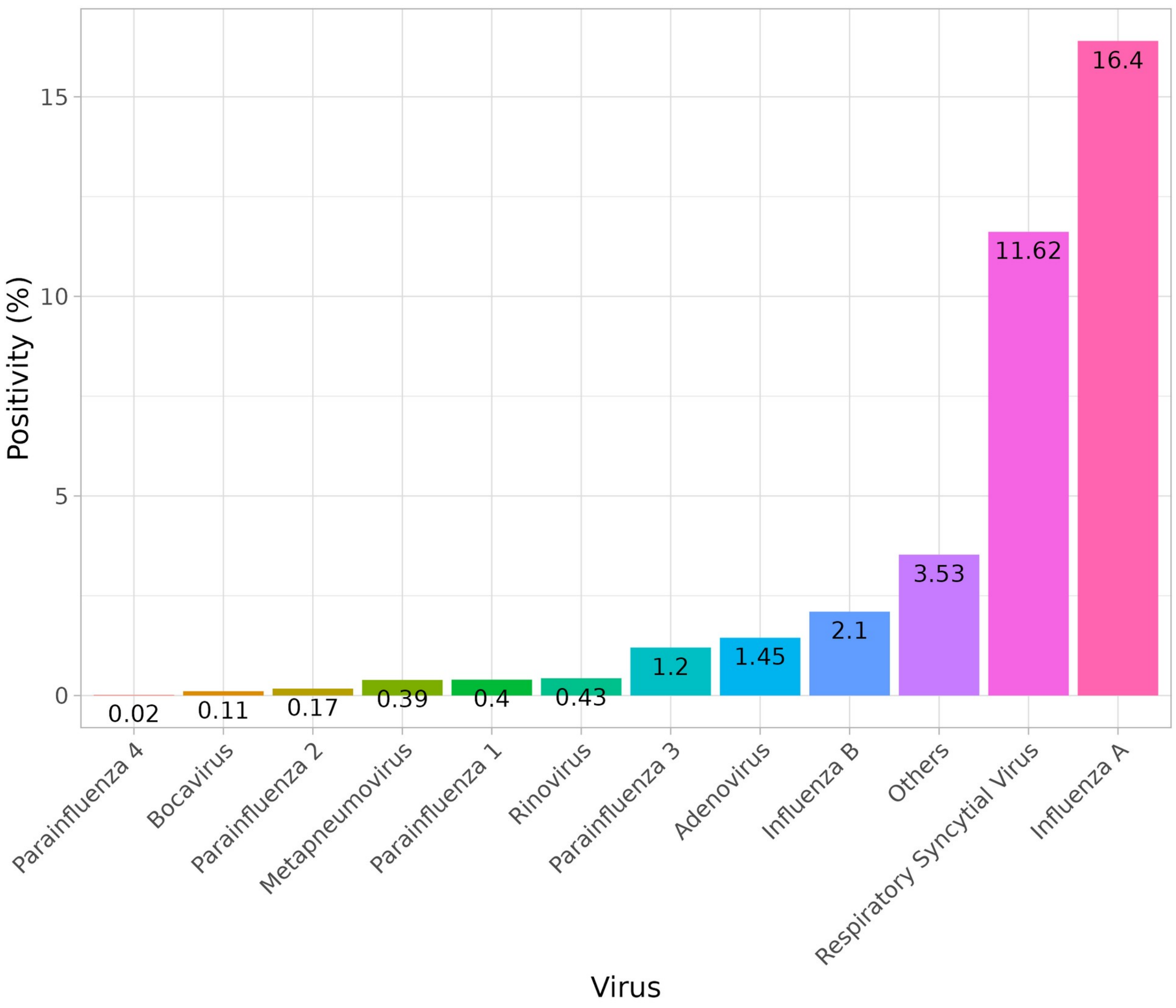

Figure S1 Positivity of respiratory viruses among tested cases of severe acute respiratory illness (SARI), Brazil, 2014-2019.

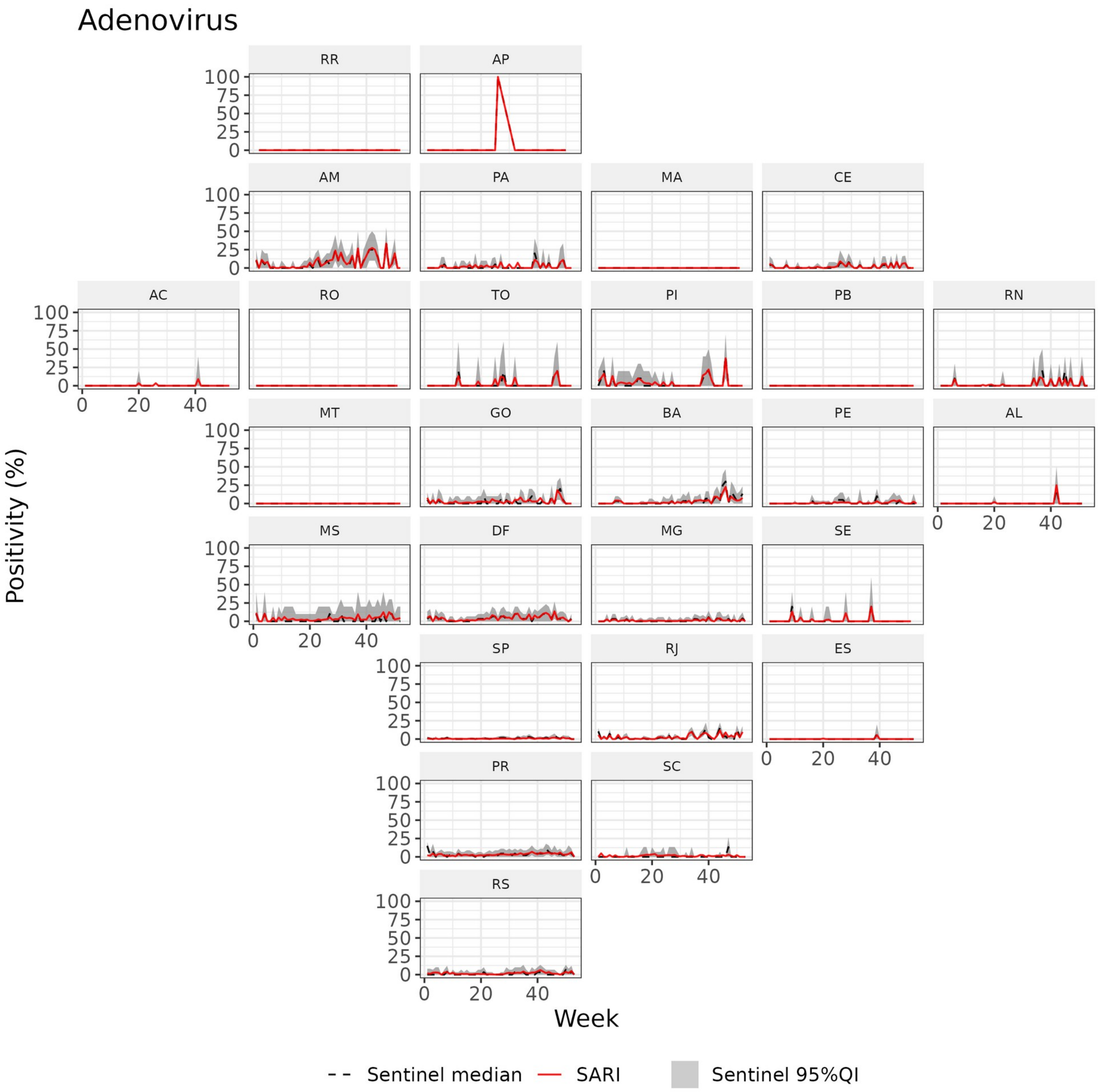

Figure S2 Adenovirus positivity in the simulated influenza-like illness sentinel surveillance (median and 95% quantile interval – 95%QI) and among tested cases of severe acute respiratory illness (SARI) by Federative Unit and epidemiological week, Brazil, 2014-2019.

Federative Units: AC: Acre State; AL: Alagoas State; AM: Amazonas State; AP: Amapá State; BA: Bahia State; CE: Ceará State; DF: Distrito Federal; ES: Espírito Santo State; GO: Goiás State; MA: Maranhão State; MG: Minas Gerais State; MS: Mato Grosso do Sul State; MT: Mato Grosso State; PA: Pará State; PB: Paraíba State; PE: Pernambuco State; PI: Piauí State; PR: Paraná State; RJ: Rio de Janeiro State; RN: Rio Grande do Norte State; RO: Rondônia State; RR: Roraima State; RS: Rio Grande do Sul State; SC: Santa Catarina State; SE: Sergipe State; SP: São Paulo State; TO: Tocantins State.

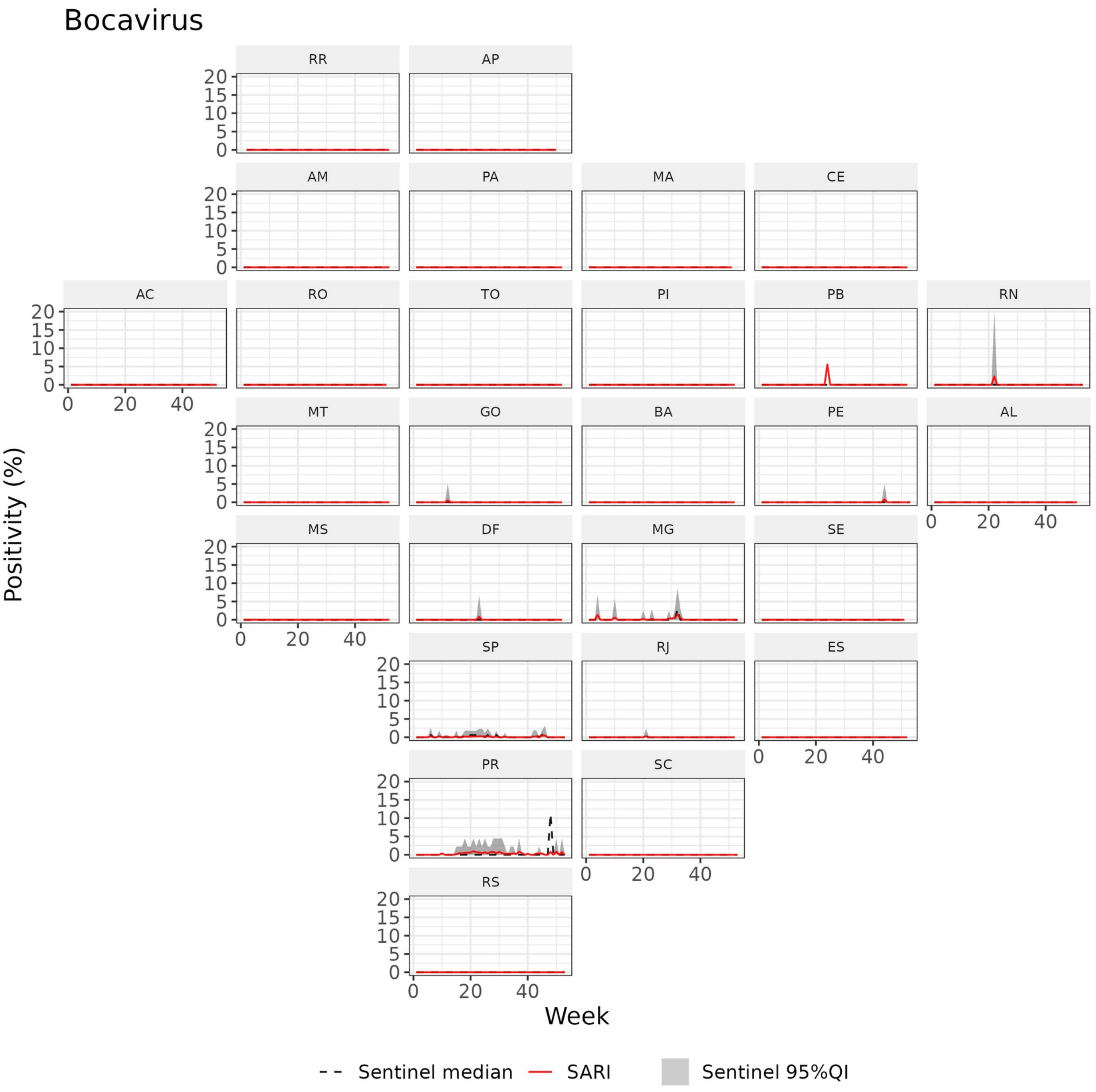

Figure S3 Bocavirus positivity in the simulated influenza-like illness sentinel surveillance (median and 95% quantile interval – 95%QI) and among tested cases of severe acute respiratory illness (SARI) by Federative Unit and epidemiological week, Brazil, 2014-2019.

Federative Units: AC: Acre State; AL: Alagoas State; AM: Amazonas State; AP: Amapá State; BA: Bahia State; CE: Ceará State; DF: Distrito Federal; ES: Espírito Santo State; GO: Goiás State; MA: Maranhão State; MG: Minas Gerais State; MS: Mato Grosso do Sul State; MT: Mato Grosso State; PA: Pará State; PB: Paraíba State; PE: Pernambuco State; PI: Piauí State; PR: Paraná State; RJ: Rio de Janeiro State; RN: Rio Grande do Norte State; RO: Rondônia State; RR: Roraima State; RS: Rio Grande do Sul State; SC: Santa Catarina State; SE: Sergipe State; SP: São Paulo State; TO: Tocantins State.

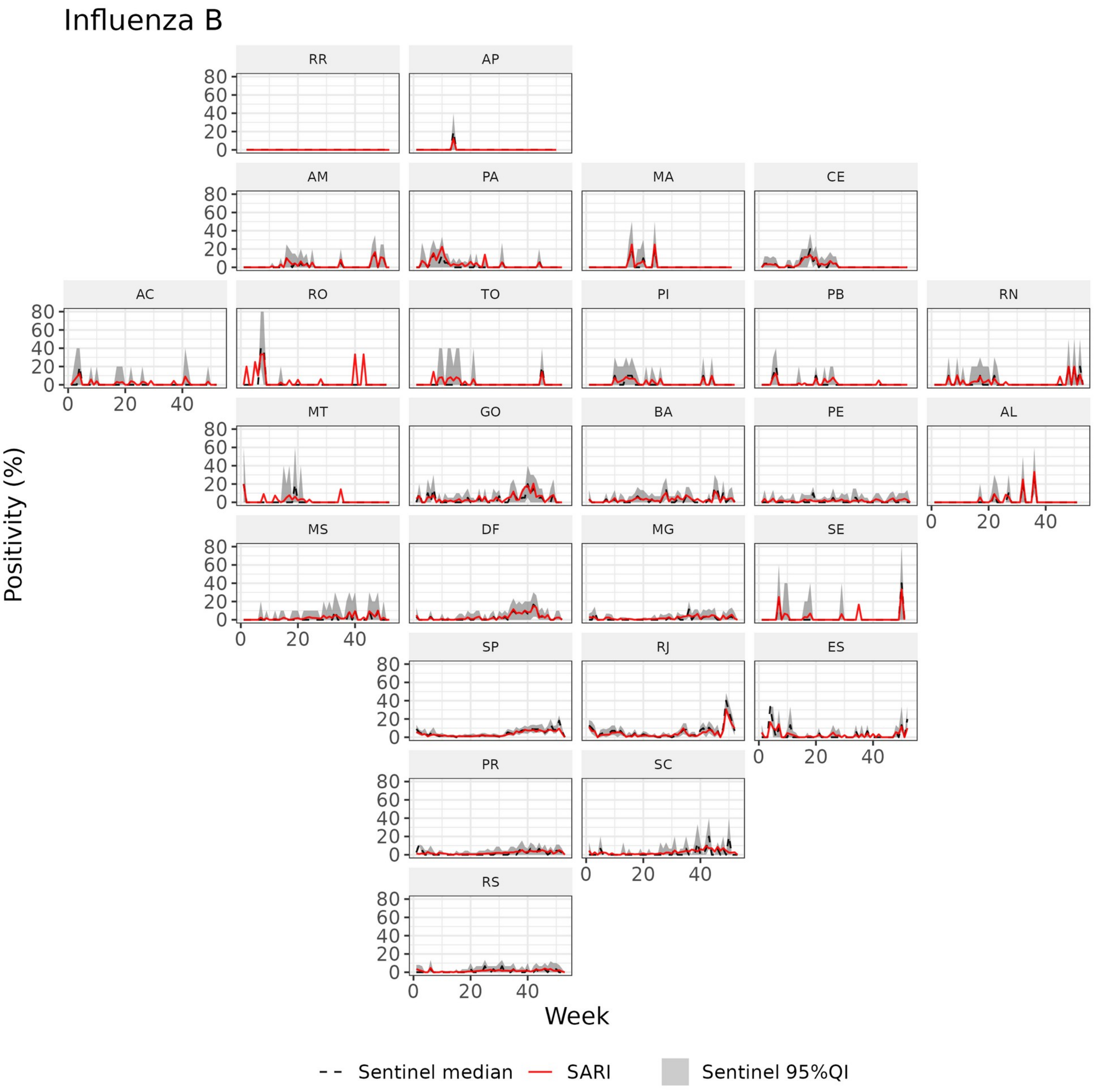

Figure S4 Influenza B positivity in the simulated influenza-like illness sentinel surveillance (median and 95% quantile interval – 95%QI) and among tested cases of severe acute respiratory illness (SARI) by Federative Unit and epidemiological week, Brazil, 2014-2019.

Federative Units: AC: Acre State; AL: Alagoas State; AM: Amazonas State; AP: Amapá State; BA: Bahia State; CE: Ceará State; DF: Distrito Federal; ES: Espírito Santo State; GO: Goiás State; MA: Maranhão State; MG: Minas Gerais State; MS: Mato Grosso do Sul State; MT: Mato Grosso State; PA: Pará State; PB: Paraíba State; PE: Pernambuco State; PI: Piauí State; PR: Paraná State; RJ: Rio de Janeiro State; RN: Rio Grande do Norte State; RO: Rondônia State; RR: Roraima State; RS: Rio Grande do Sul State; SC: Santa Catarina State; SE: Sergipe State; SP: São Paulo State; TO: Tocantins State.

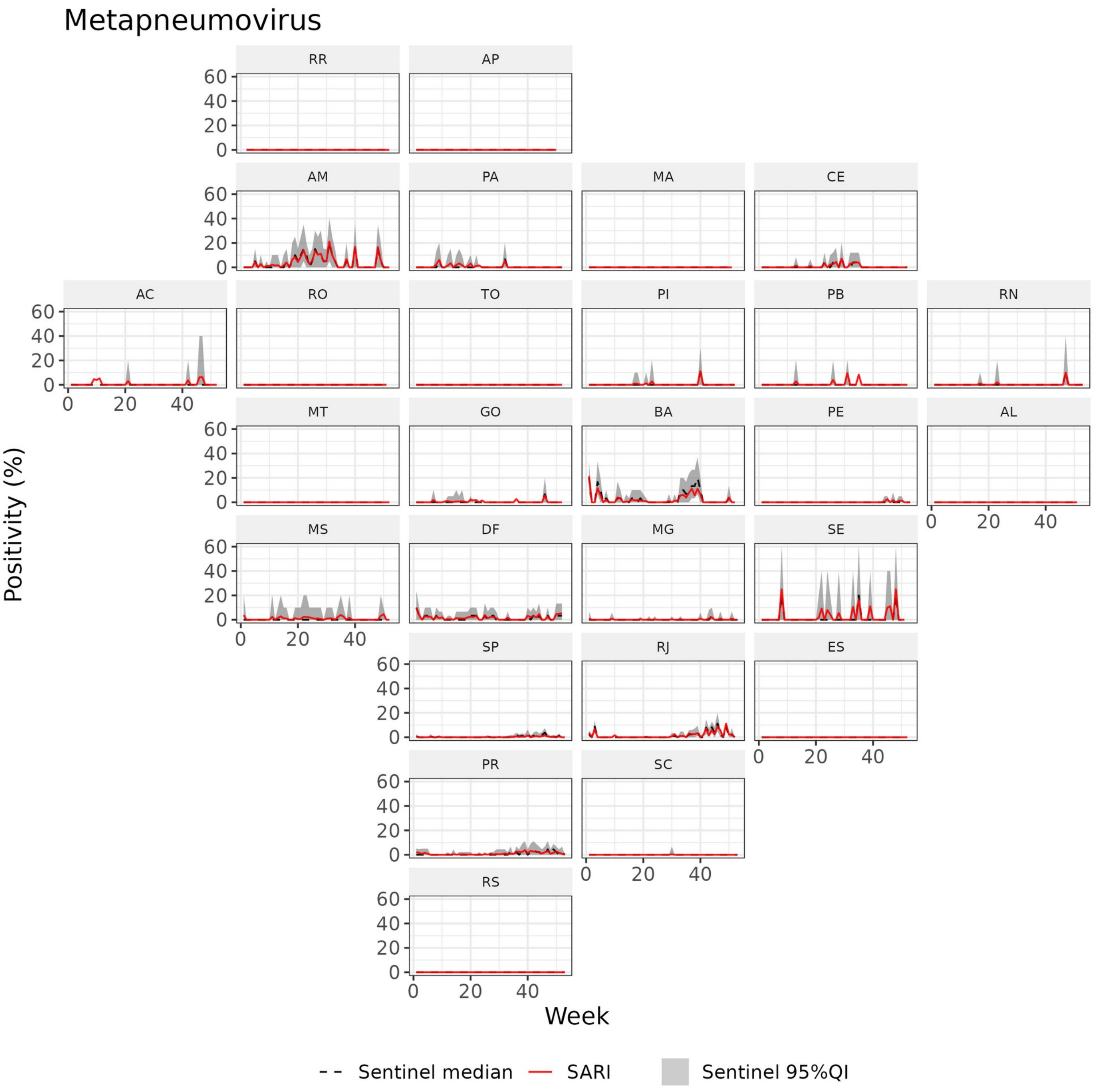

Figure S5 Metapneumovirus positivity in the simulated influenza-like illness sentinel surveillance (median and 95% quantile interval – 95%QI) and among tested cases of severe acute respiratory illness (SARI) by Federative Unit and epidemiological week, Brazil, 2014-2019.

Federative Units: AC: Acre State; AL: Alagoas State; AM: Amazonas State; AP: Amapá State; BA: Bahia State; CE: Ceará State; DF: Distrito Federal; ES: Espírito Santo State; GO: Goiás State; MA: Maranhão State; MG: Minas Gerais State; MS: Mato Grosso do Sul State; MT: Mato Grosso State; PA: Pará State; PB: Paraíba State; PE: Pernambuco State; PI: Piauí State; PR: Paraná State; RJ: Rio de Janeiro State; RN: Rio Grande do Norte State; RO: Rondônia State; RR: Roraima State; RS: Rio Grande do Sul State; SC: Santa Catarina State; SE: Sergipe State; SP: São Paulo State; TO: Tocantins State.

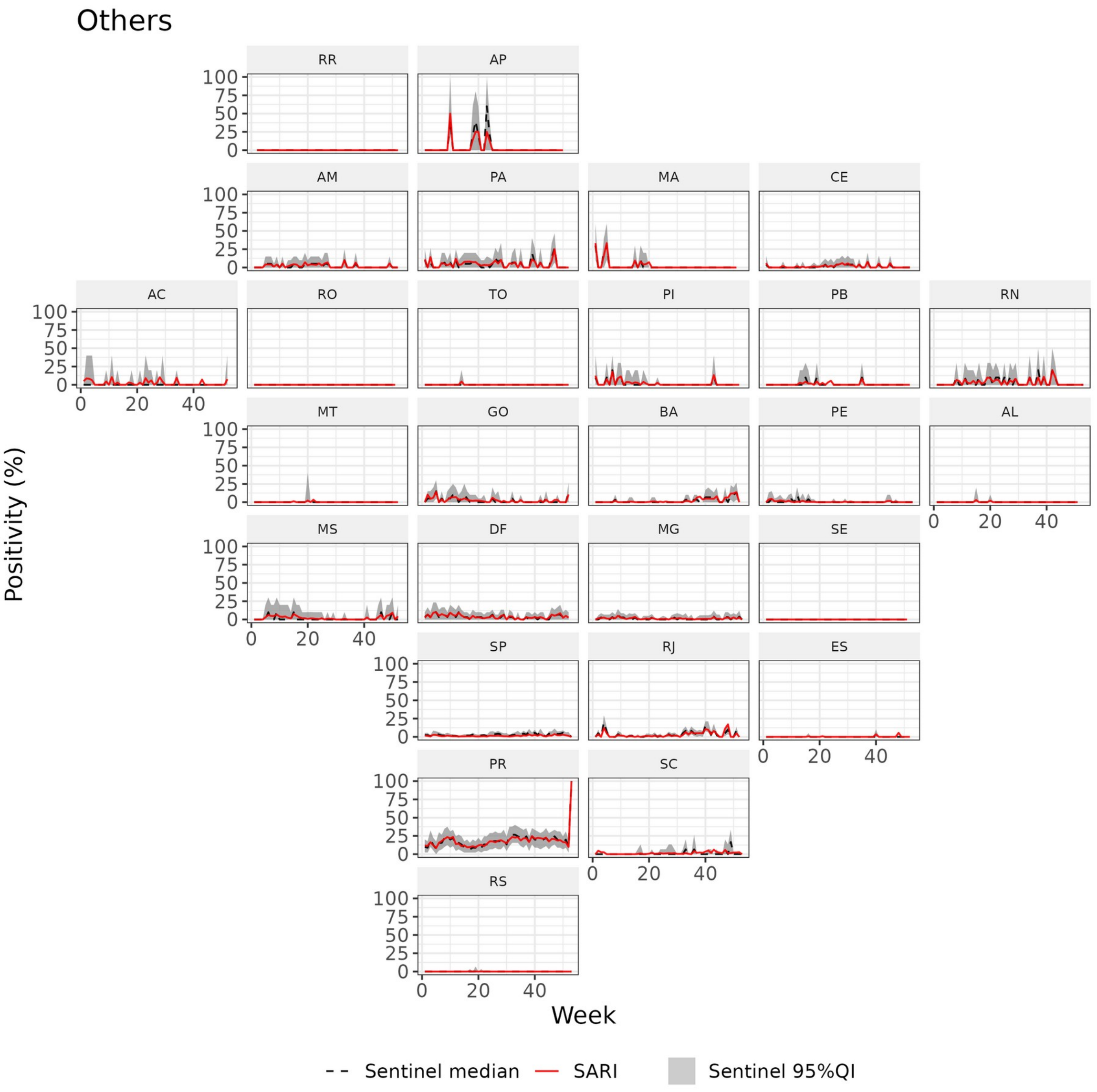

Figure S6 Other viruses positivity in the simulated influenza-like illness sentinel surveillance (median and 95% quantile interval – 95%QI) and among tested cases of severe acute respiratory illness (SARI) by Federative Unit and epidemiological week, Brazil, 2014-2019.

Federative Units: AC: Acre State; AL: Alagoas State; AM: Amazonas State; AP: Amapá State; BA: Bahia State; CE: Ceará State; DF: Distrito Federal; ES: Espírito Santo State; GO: Goiás State; MA: Maranhão State; MG: Minas Gerais State; MS: Mato Grosso do Sul State; MT: Mato Grosso State; PA: Pará State; PB: Paraíba State; PE: Pernambuco State; PI: Piauí State; PR: Paraná State; RJ: Rio de Janeiro State; RN: Rio Grande do Norte State; RO: Rondônia State; RR: Roraima State; RS: Rio Grande do Sul State; SC: Santa Catarina State; SE: Sergipe State; SP: São Paulo State; TO: Tocantins State.

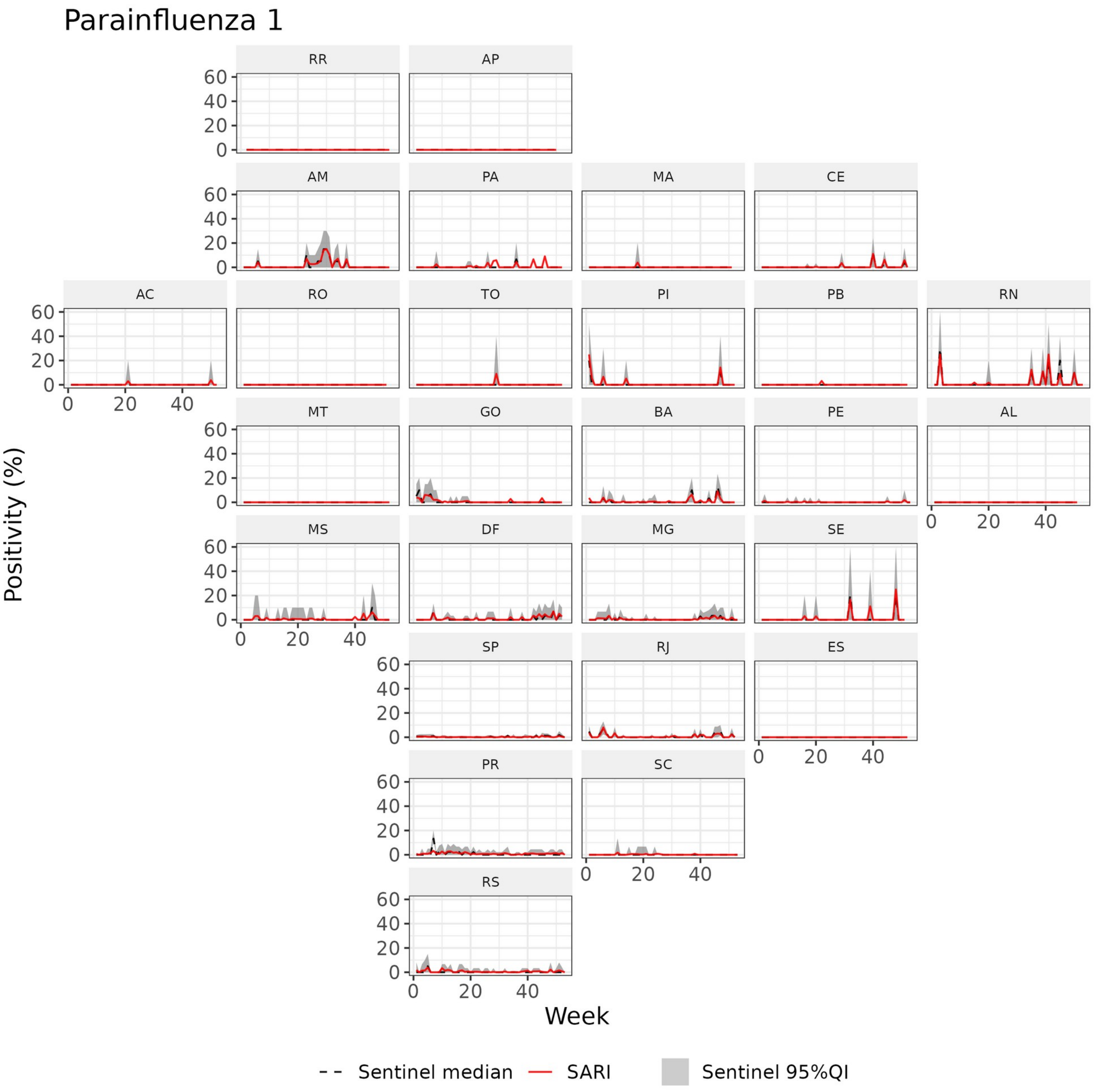

Figure S7 Parainfluenza 1 positivity in the simulated influenza-like illness sentinel surveillance (median and 95% quantile interval – 95QI) and among tested cases of severe acute respiratory illness (SARI) by Federative Unit and epidemiological week, Brazil, 2014-2019.

Federative Units: AC: Acre State; AL: Alagoas State; AM: Amazonas State; AP: Amapá State; BA: Bahia State; CE: Ceará State; DF: Distrito Federal; ES: Espírito Santo State; GO: Goiás State; MA: Maranhão State; MG: Minas Gerais State; MS: Mato Grosso do Sul State; MT: Mato Grosso State; PA: Pará State; PB: Paraíba State; PE: Pernambuco State; PI: Piauí State; PR: Paraná State; RJ: Rio de Janeiro State; RN: Rio Grande do Norte State; RO: Rondônia State; RR: Roraima State; RS: Rio Grande do Sul State; SC: Santa Catarina State; SE: Sergipe State; SP: São Paulo State; TO: Tocantins State.

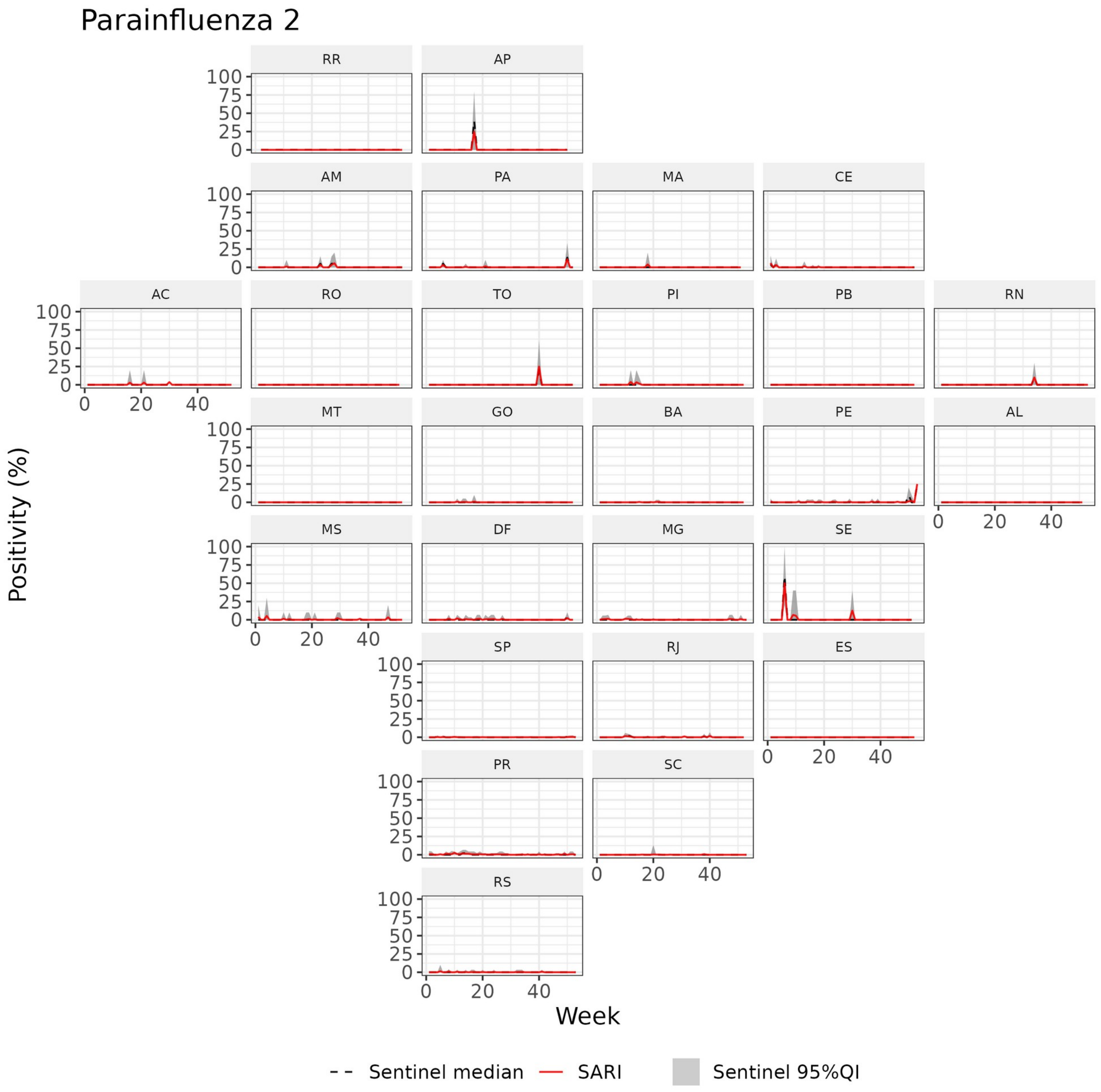

Figure S8 Parainfluenza 2 positivity in the simulated influenza-like illness sentinel surveillance (median and 95% quantile interval – 95%QI) and among tested cases of severe acute respiratory illness (SARI) by Federative Unit and epidemiological week, Brazil, 2014-2019.

Federative Units: AC: Acre State; AL: Alagoas State; AM: Amazonas State; AP: Amapá State; BA: Bahia State; CE: Ceará State; DF: Distrito Federal; ES: Espírito Santo State; GO: Goiás State; MA: Maranhão State; MG: Minas Gerais State; MS: Mato Grosso do Sul State; MT: Mato Grosso State; PA: Pará State; PB: Paraíba State; PE: Pernambuco State; PI: Piauí State; PR: Paraná State; RJ: Rio de Janeiro State; RN: Rio Grande do Norte State; RO: Rondônia State; RR: Roraima State; RS: Rio Grande do Sul State; SC: Santa Catarina State; SE: Sergipe State; SP: São Paulo State; TO: Tocantins State.

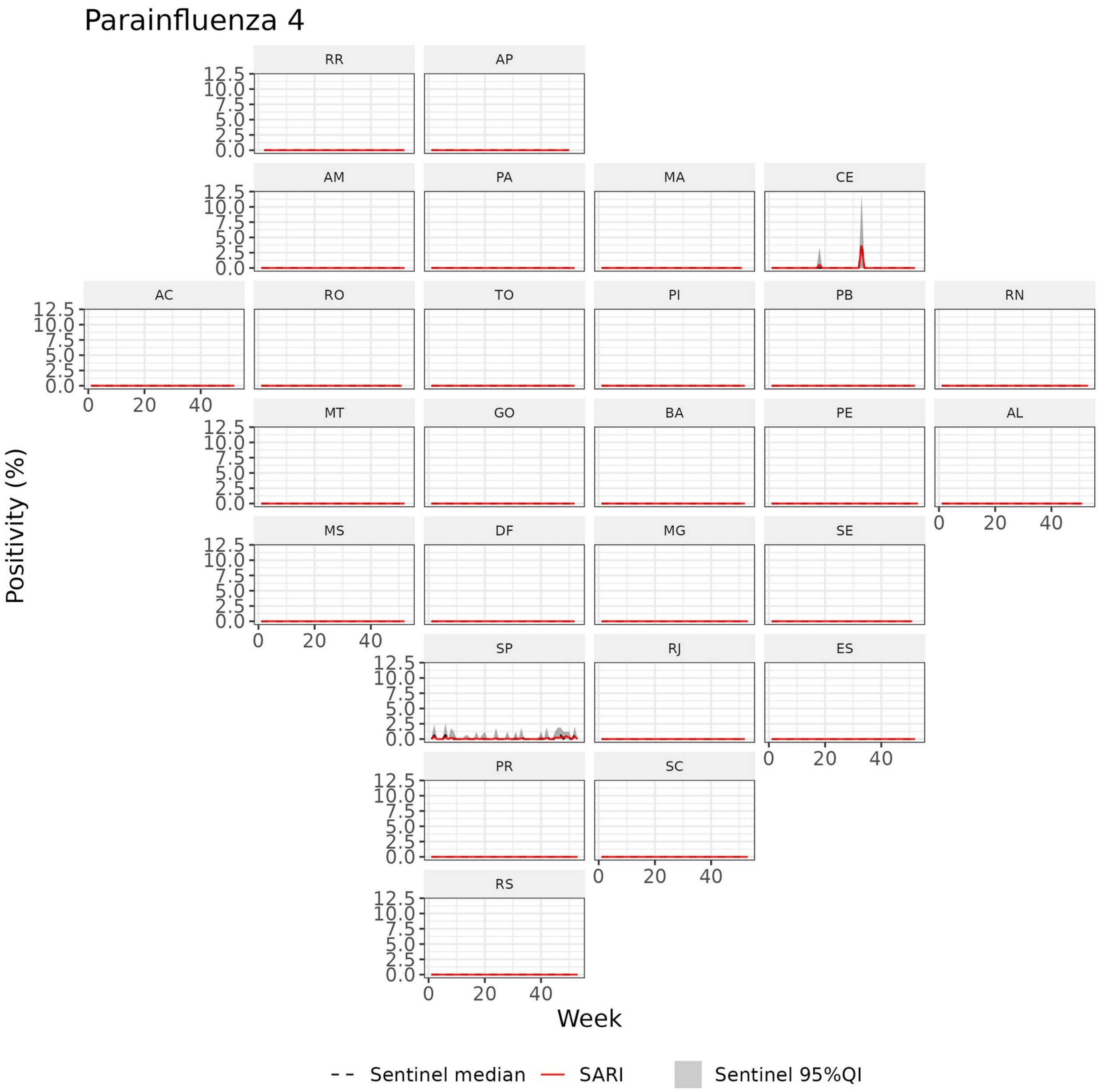

Figure S9 Parainfluenza 4 positivity in the simulated influenza-like illness sentinel surveillance (median and 95% quantile interval – 95%QI) and among tested cases of severe acute respiratory illness (SARI) by Federative Unit and epidemiological week, Brazil, 2014-2019.

Federative Units: AC: Acre State; AL: Alagoas State; AM: Amazonas State; AP: Amapá State; BA: Bahia State; CE: Ceará State; DF: Distrito Federal; ES: Espírito Santo State; GO: Goiás State; MA: Maranhão State; MG: Minas Gerais State; MS: Mato Grosso do Sul State; MT: Mato Grosso State; PA: Pará State; PB: Paraíba State; PE: Pernambuco State; PI: Piauí State; PR: Paraná State; RJ: Rio de Janeiro State; RN: Rio Grande do Norte State; RO: Rondônia State; RR: Roraima State; RS: Rio Grande do Sul State; SC: Santa Catarina State; SE: Sergipe State; SP: São Paulo State; TO: Tocantins State.

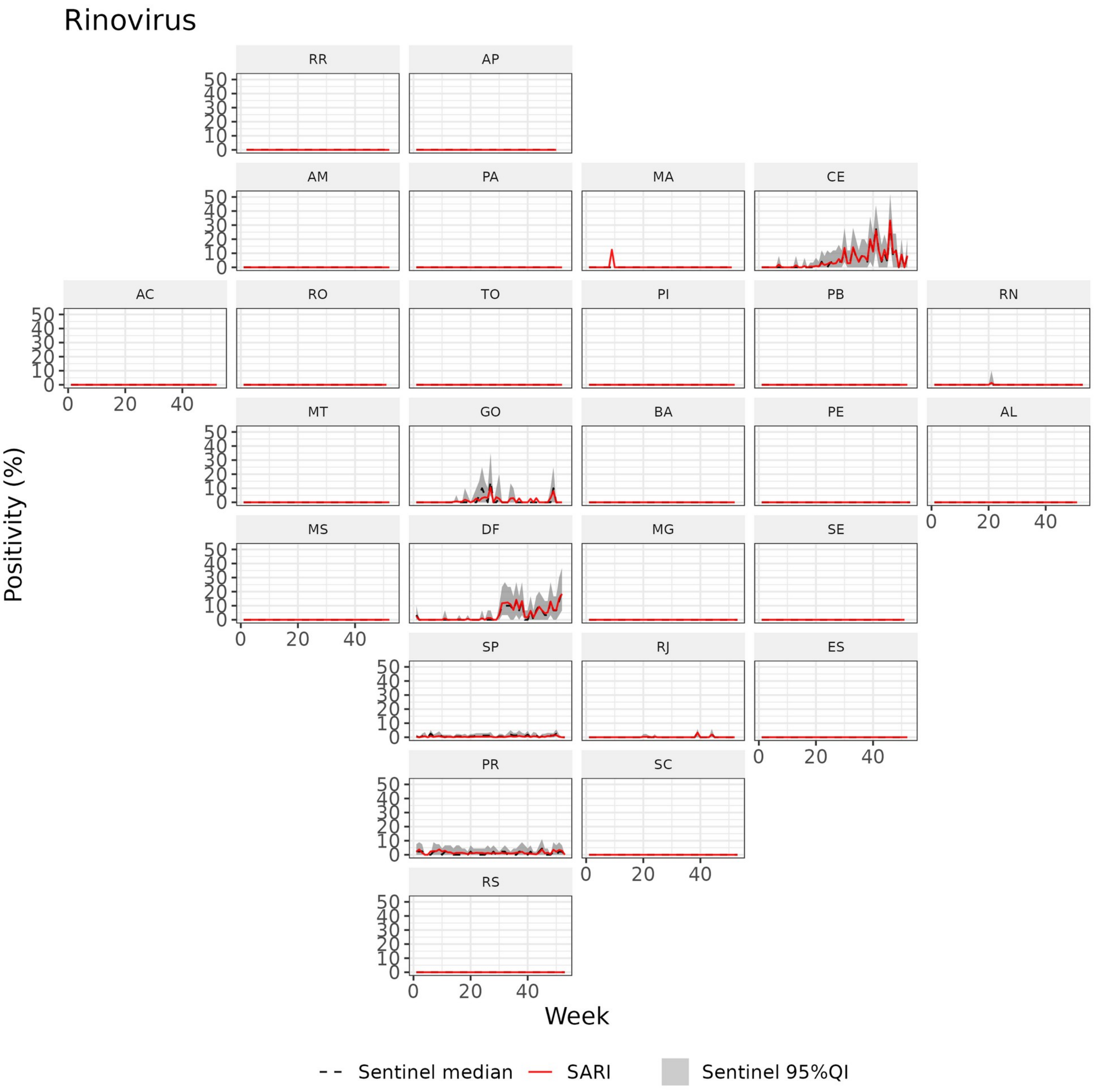

Figure S10 Rinovirus positivity in the simulated influenza-like illness sentinel surveillance (median and 95% quantile interval – 95%QI) and among tested cases of severe acute respiratory illness (SARI) by Federative Unit and epidemiological week, Brazil, 2014-2019.

Federative Units: AC: Acre State; AL: Alagoas State; AM: Amazonas State; AP: Amapá State; BA: Bahia State; CE: Ceará State; DF: Distrito Federal; ES: Espírito Santo State; GO: Goiás State; MA: Maranhão State; MG: Minas Gerais State; MS: Mato Grosso do Sul State; MT: Mato Grosso State; PA: Pará State; PB: Paraíba State; PE: Pernambuco State; PI: Piauí State; PR: Paraná State; RJ: Rio de Janeiro State; RN: Rio Grande do Norte State; RO: Rondônia State; RR: Roraima State; RS: Rio Grande do Sul State; SC: Santa Catarina State; SE: Sergipe State; SP: São Paulo State; TO: Tocantins State.

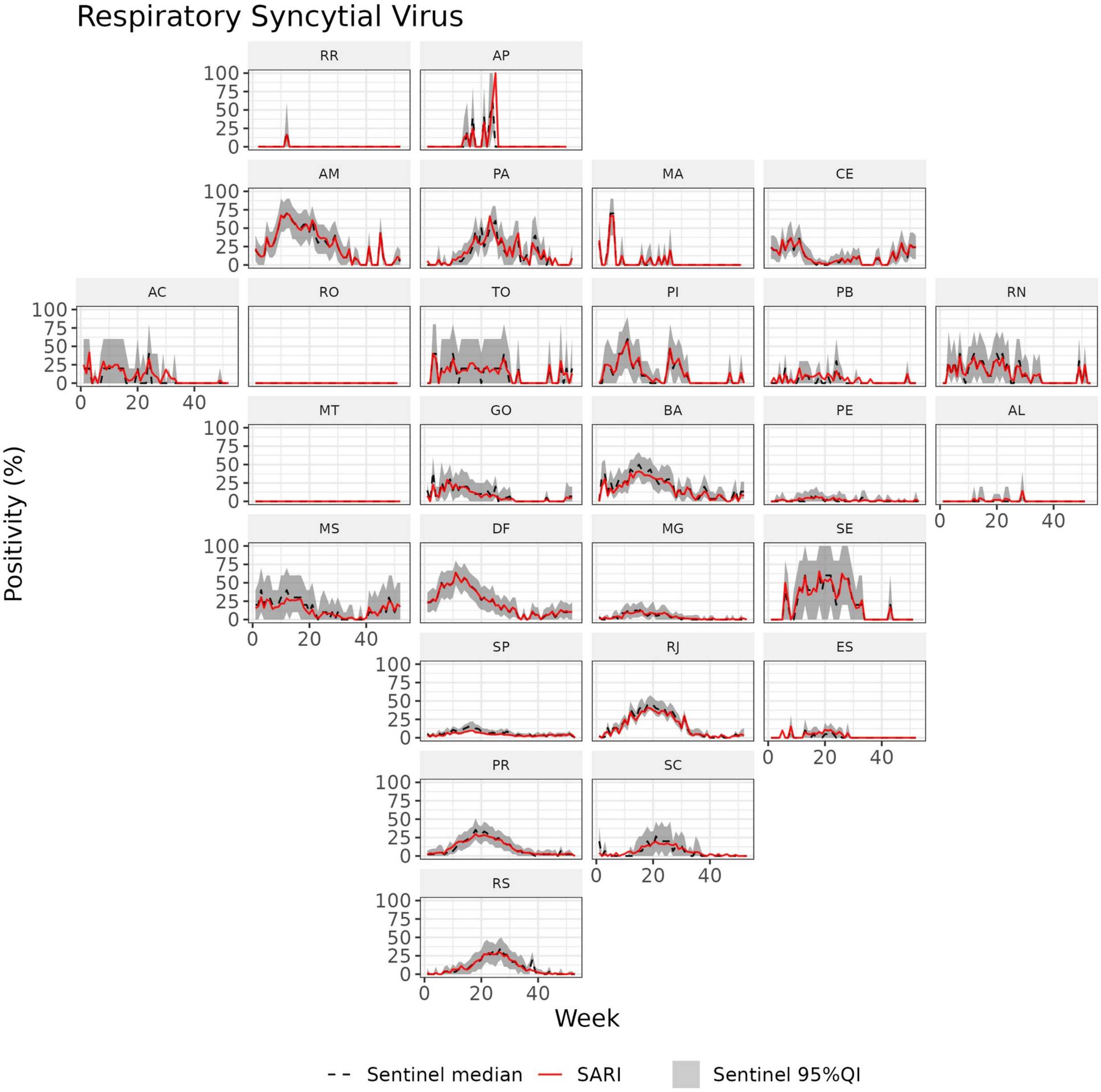

Figure S11 Respiratory syncytial virus positivity in the simulated influenza-like illness sentinel surveillance (median and 95% quantile interval – 95%QI) and among tested cases of severe acute respiratory illness (SARI) by Federative Unit and epidemiological week, Brazil, 2014-2019.

Federative Units: AC: Acre State; AL: Alagoas State; AM: Amazonas State; AP: Amapá State; BA: Bahia State; CE: Ceará State; DF: Distrito Federal; ES: Espírito Santo State; GO: Goiás State; MA: Maranhão State; MG: Minas Gerais State; MS: Mato Grosso do Sul State; MT: Mato Grosso State; PA: Pará State; PB: Paraíba State; PE: Pernambuco State; PI: Piauí State; PR: Paraná State; RJ: Rio de Janeiro State; RN: Rio Grande do Norte State; RO: Rondônia State; RR: Roraima State; RS: Rio Grande do Sul State; SC: Santa Catarina State; SE: Sergipe State; SP: São Paulo State; TO: Tocantins State.
