# Supplementary material for: Evaluation of the design of the influenza-like illness sentinel surveillance system in Brazil
Source: Cad Saude Publica. 2024 Jul 29;40(6):e00028823. doi: 10.1590/0102-311XEN028823 (PMC11321611; doi:10.1590/0102-311XEN028823)
Supplement: Supplementary file 2 [file 1678-4464-csp-40-06-EN028823-s-pt.pdf]

Tabela S1 Número e nome dos municípios que estariam incluídos na rede de vigilância sentinela de síndrome gripal por número de unidades sentinelas no município e número de amostras semanais preconizadas para coleta, segundo os critérios na *Portaria nº 183* do Ministério da Saúde.

Número de unidades sentinelas preconizadas no município	Número de amostras semanais preconizadas	Número de municípios elegíveis	Nome dos municípios elegíveis para compor a rede sentinela
1	5	46	Rio Branco/AC, Macapá/AP, Caucaia/CE, Cariacica/ES, Serra/ES, Vila Velha/ES, Vitória/ES, Aparecida De Goiânia/GO, Betim/MG, Contagem/MG, Ribeirão Das Neves/MG, Cuiabá/MT, Ananindeua/PA, Jaboatão Dos Guararapes/PE, Olinda/PE, Paulista/PE, Cascavel/PR, Londrina/PR, Maringá/PR, Ponta Grossa/PR, São José Dos Pinhais/PR, Belford Roxo/RJ, Duque De Caxias/RJ, Niterói/RJ, Nova Iguacu/RJ, São Gonçalo/RJ, São João De Meriti/RJ, Porto Velho/RO, Boa Vista/RR, Canoas/RS, Caxias Do Sul/RS, Pelotas/RS, Blumenau/SC, Florianópolis/SC, Joinville/SC, Aracaju/SE, Carapicuíba/SP, Diadema/SP, Guarulhos/SP, Itaquaquecetuba/SP, Mauá/SP, Mogi Das Cruzes/SP, Osasco/SP, Santo André/SP, São Bernardo Do Campo/SP, Palmas/TO
2	10	6	Maceió/AL, São Luís/MA, Campo Grande/MS, João Pessoa/PB, Teresina/PI, Natal/RN
3	15	4	Goiânia/GO, Belém/PA, Recife/PE, Porto Alegre/RS
4	20	2	Manaus/AM, Curitiba/PR
5	25	2	Fortaleza/CE, Belo Horizonte/MG
6	30	2	Salvador/BA, Brasília/DF
13	65	1	Rio De Janeiro/RJ
25	125	1	São Paulo/SP

Unidades Federativas: AC: Acre; AL: Alagoas; AM: Amazonas; AP: Amapá; BA: Bahia; CE: Ceará; DF: Distrito Federal; ES: Espírito Santo; GO: Goiás; MA: Maranhão; MG: Minas Gerais; MS: Mato Grosso do Sul; MT: Mato Grosso; PA: Pará; PB: Paraíba; PE: Pernambuco; PI: Piauí; PR: Paraná; RJ: Rio de Janeiro; RN: Rio Grande do Norte; RO: Rondônia; RR: Roraima; RS: Rio Grande do Sul; SC: Santa Catarina; SE: Sergipe; SP: São Paulo; TO: Tocantins.

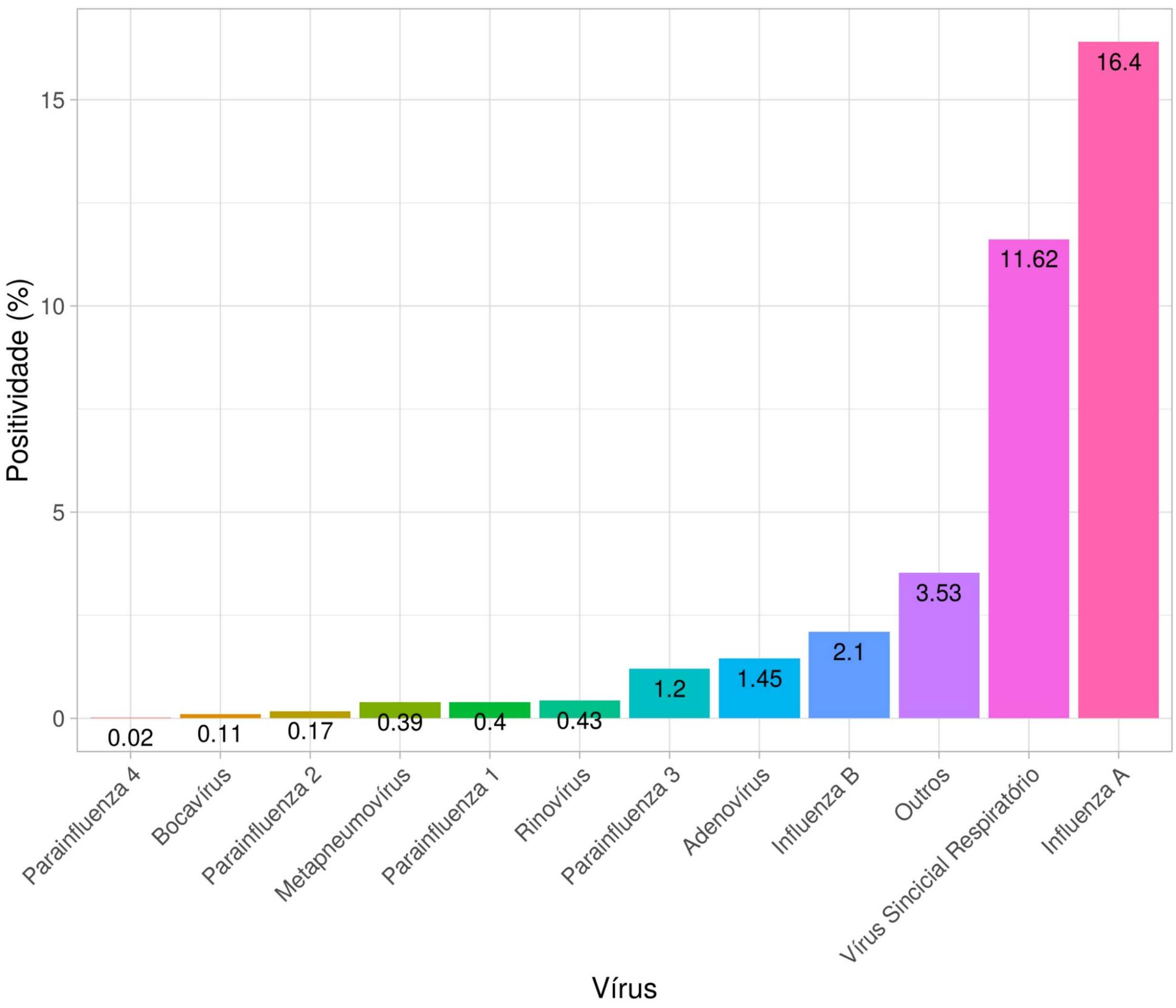

Figura S1 Positividade de vírus respiratórios entre os casos testados de síndrome respiratória aguda grave (SRAG), Brasil, 2014-2019.

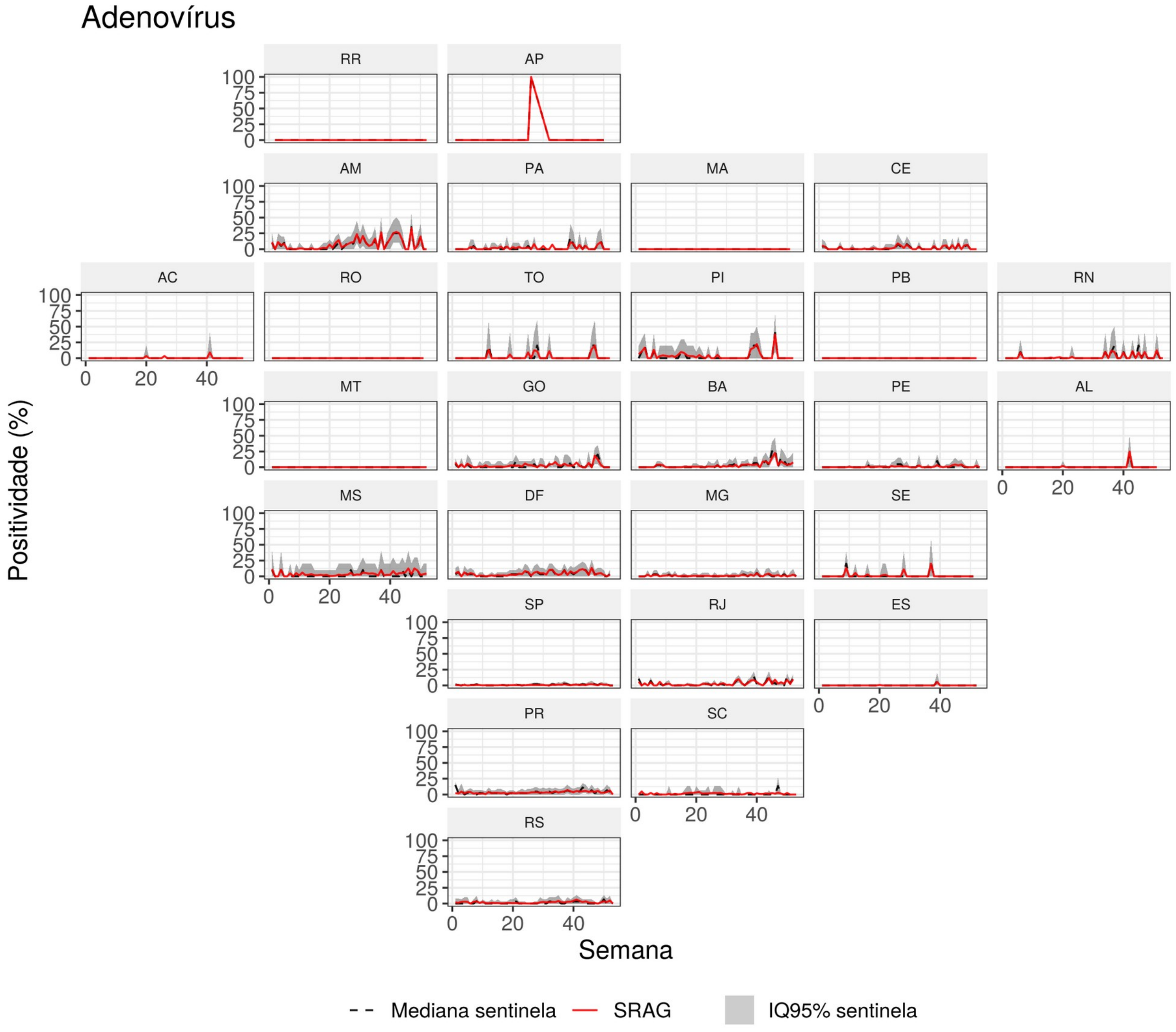

Figura S2 Positividade de adenovírus na vigilância sentinela de síndrome gripal simulada (mediana e intervalo quantílico de 95% – IQ95%) e nos casos testados de síndrome respiratória aguda grave (SRAG) por Unidade Federativa e semana epidemiológica, Brasil, 2014-2019.

Unidades Federativas: AC: Acre; AL: Alagoas; AM: Amazonas; AP: Amapá; BA: Bahia; CE: Ceará; DF: Distrito Federal; ES: Espírito Santo; GO: Goiás; MA: Maranhão; MG: Minas Gerais; MS: Mato Grosso do Sul; MT: Mato Grosso; PA: Pará; PB: Paraíba; PE: Pernambuco; PI: Piauí; PR: Paraná; RJ: Rio de Janeiro; RN: Rio Grande do Norte; RO: Rondônia; RR: Roraima; RS: Rio Grande do Sul; SC: Santa Catarina; SE: Sergipe; SP: São Paulo; TO: Tocantins.

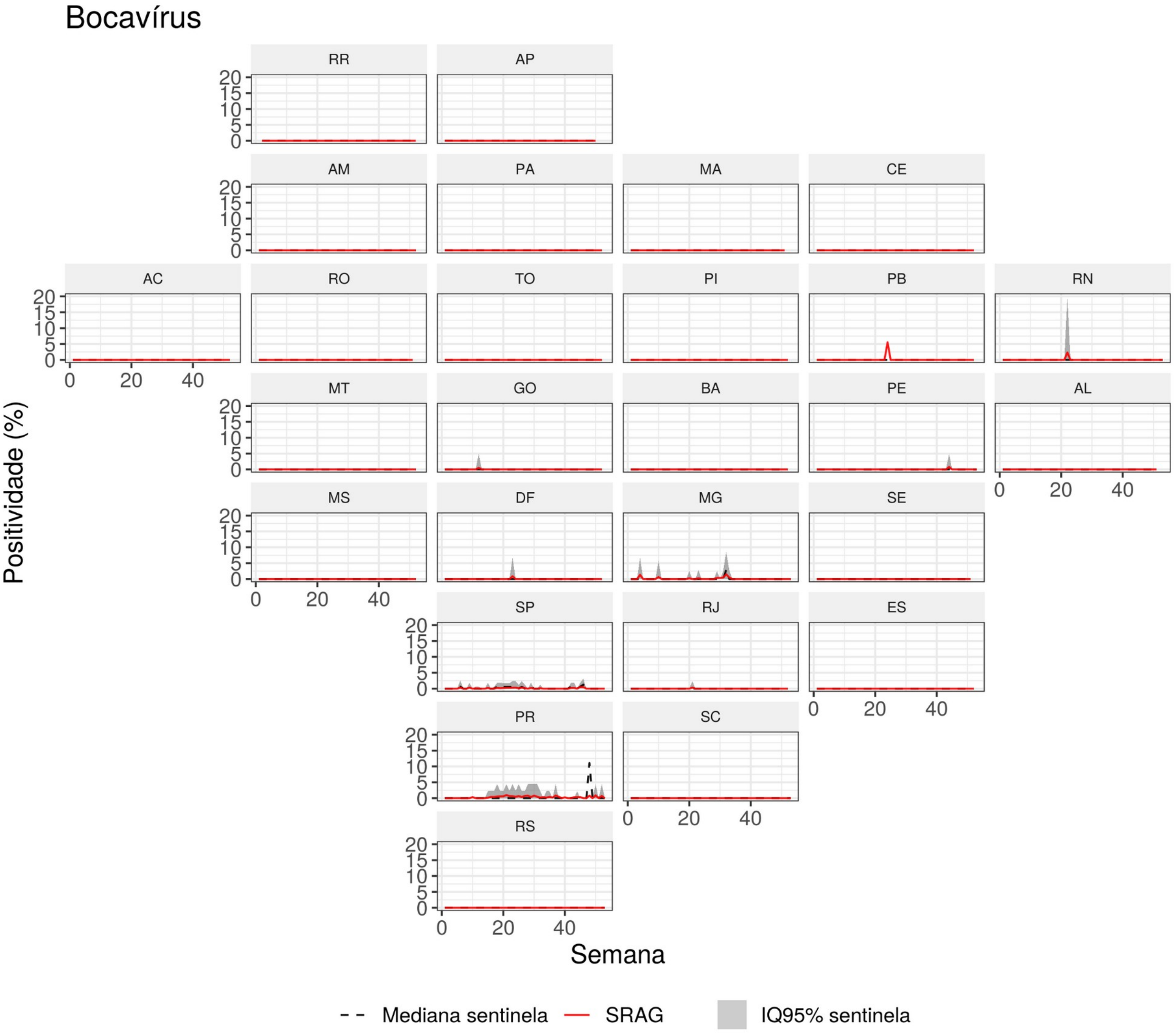

Figura S3 Positividade de bocavírus na vigilância sentinela de síndrome gripal simulada (mediana e intervalo quantílico de 95% – IQ95%) e nos casos testados de síndrome respiratória aguda grave (SRAG) por Unidade Federativa e semana epidemiológica, Brasil, 2014-2019.

Unidades Federativas: AC: Acre; AL: Alagoas; AM: Amazonas; AP: Amapá; BA: Bahia; CE: Ceará; DF: Distrito Federal; ES: Espírito Santo; GO: Goiás; MA: Maranhão; MG: Minas Gerais; MS: Mato Grosso do Sul; MT: Mato Grosso; PA: Pará; PB: Paraíba; PE: Pernambuco; PI: Piauí; PR: Paraná; RJ: Rio de Janeiro; RN: Rio Grande do Norte; RO: Rondônia; RR: Roraima; RS: Rio Grande do Sul; SC: Santa Catarina; SE: Sergipe; SP: São Paulo; TO: Tocantins.

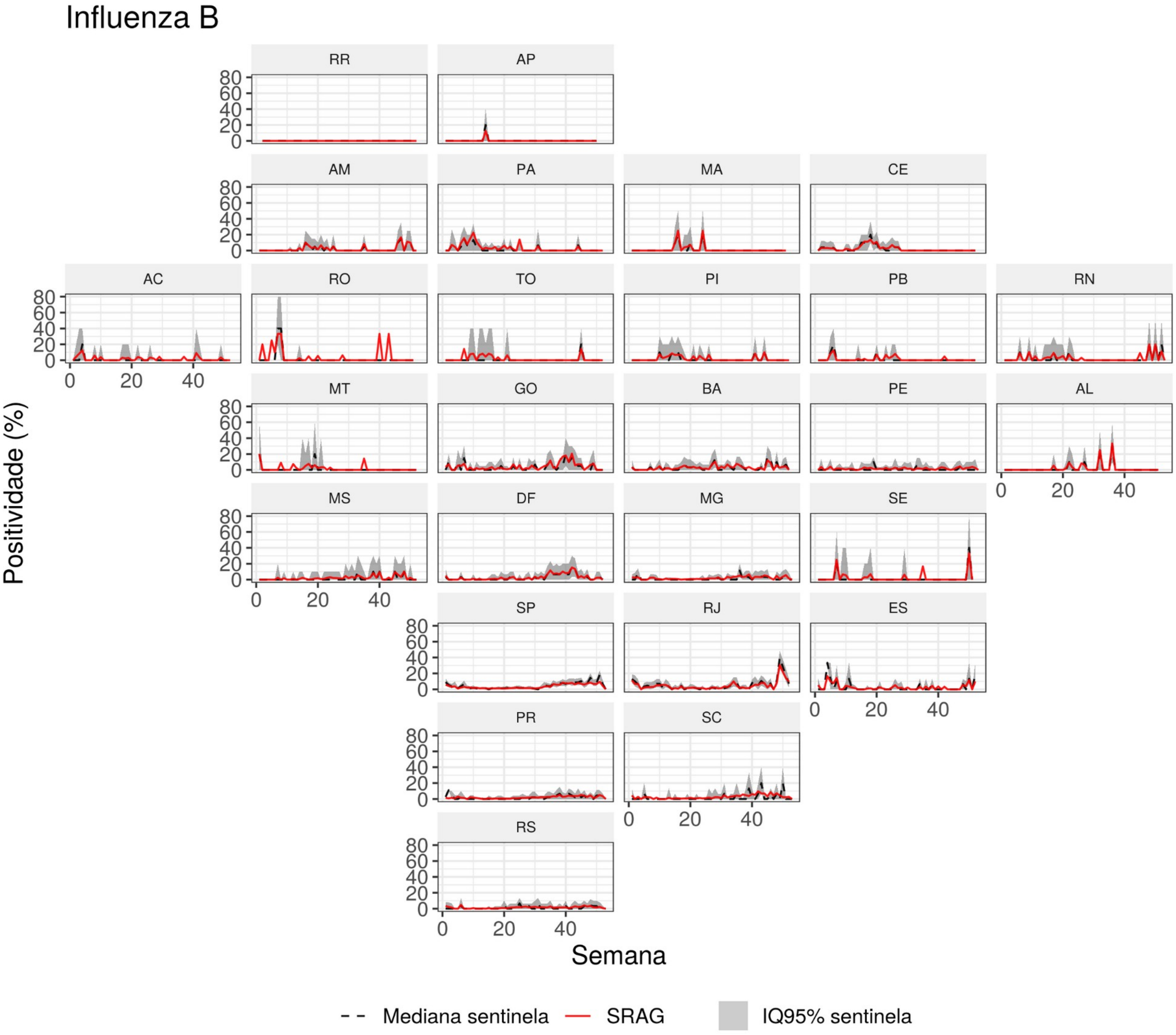

Figura S4 Positividade de influenza B na vigilância sentinela de síndrome gripal simulada (mediana e intervalo quantílico de 95% – IQ95%) e nos casos testados de síndrome respiratória aguda grave (SRAG) por Unidade Federativa e semana epidemiológica, Brasil, 2014-2019.

Unidades Federativas: AC: Acre; AL: Alagoas; AM: Amazonas; AP: Amapá; BA: Bahia; CE: Ceará; DF: Distrito Federal; ES: Espírito Santo; GO: Goiás; MA: Maranhão; MG: Minas Gerais; MS: Mato Grosso do Sul; MT: Mato Grosso; PA: Pará; PB: Paraíba; PE: Pernambuco; PI: Piauí; PR: Paraná; RJ: Rio de Janeiro; RN: Rio Grande do Norte; RO: Rondônia; RR: Roraima; RS: Rio Grande do Sul; SC: Santa Catarina; SE: Sergipe; SP: São Paulo; TO: Tocantins.

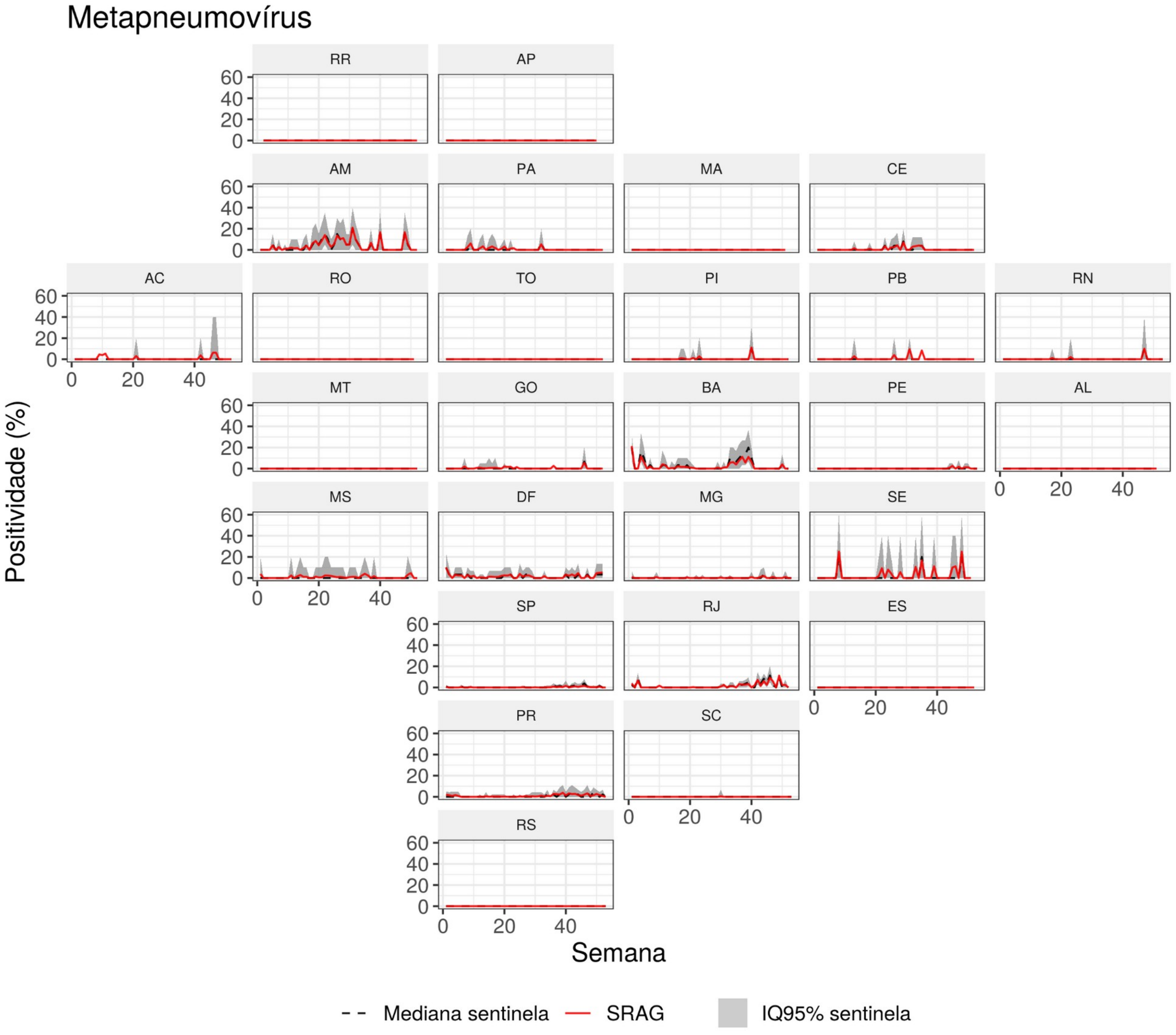

Figura S5 Positividade de metapneumovírus na vigilância sentinela de síndrome gripal simulada (mediana e intervalo quantílico de 95% – IQ95%) e nos casos testados de síndrome respiratória aguda grave (SRAG) por Unidade Federativa e semana epidemiológica, Brasil, 2014-2019.

Unidades Federativas: AC: Acre; AL: Alagoas; AM: Amazonas; AP: Amapá; BA: Bahia; CE: Ceará; DF: Distrito Federal; ES: Espírito Santo; GO: Goiás; MA: Maranhão; MG: Minas Gerais; MS: Mato Grosso do Sul; MT: Mato Grosso; PA: Pará; PB: Paraíba; PE: Pernambuco; PI: Piauí; PR: Paraná; RJ: Rio de Janeiro; RN: Rio Grande do Norte; RO: Rondônia; RR: Roraima; RS: Rio Grande do Sul; SC: Santa Catarina; SE: Sergipe; SP: São Paulo; TO: Tocantins.

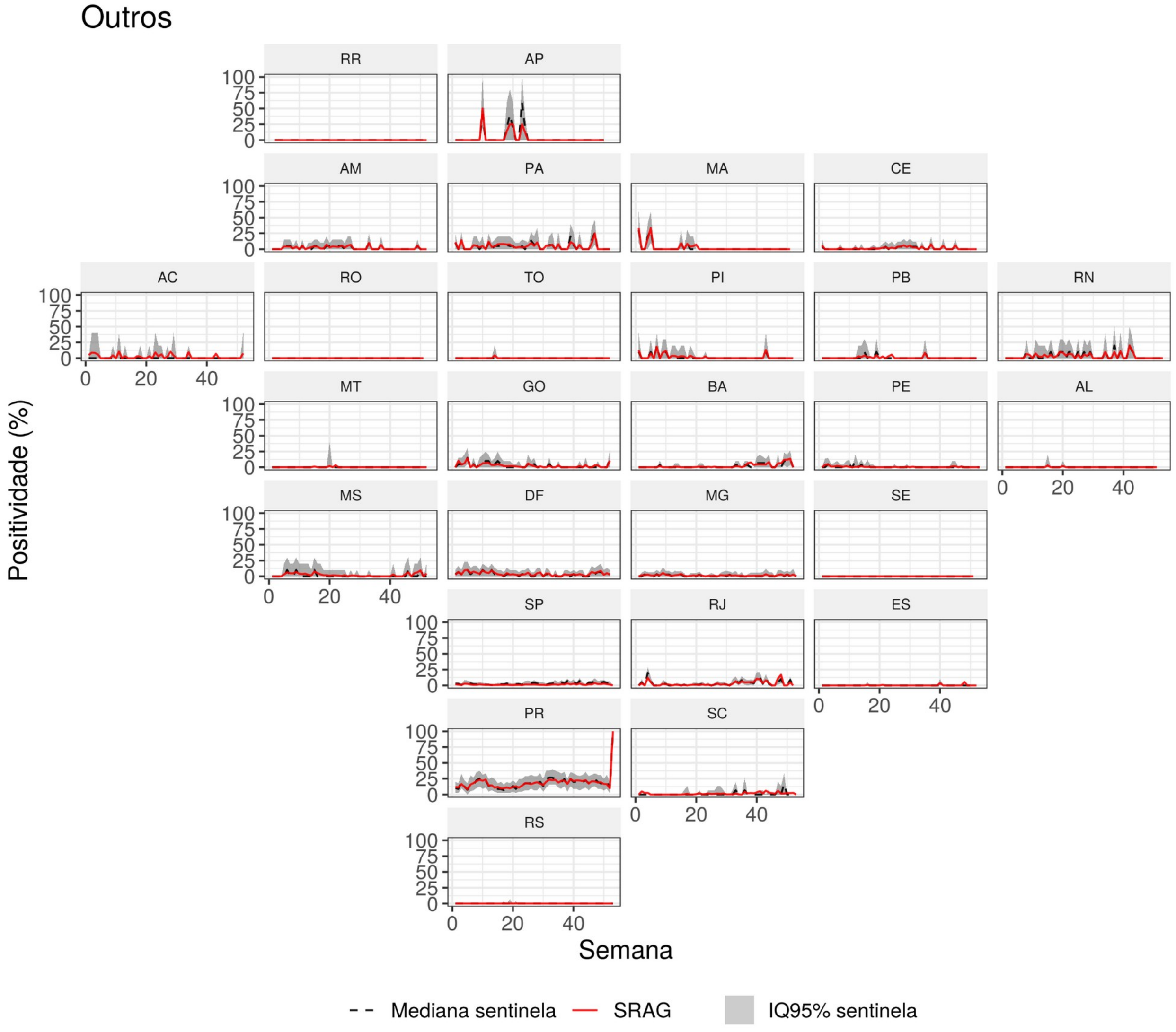

Figura S6 Positividade de outros vírus respiratórios na vigilância sentinela de síndrome gripal simulada (mediana e intervalo quantílico de 95% – IQ95%) e nos casos testados de síndrome respiratória aguda grave (SRAG) por Unidade Federativa e semana epidemiológica, Brasil, 2014-2019.

Unidades Federativas: AC: Acre; AL: Alagoas; AM: Amazonas; AP: Amapá; BA: Bahia; CE: Ceará; DF: Distrito Federal; ES: Espírito Santo; GO: Goiás; MA: Maranhão; MG: Minas Gerais; MS: Mato Grosso do Sul; MT: Mato Grosso; PA: Pará; PB: Paraíba; PE: Pernambuco; PI: Piauí; PR: Paraná; RJ: Rio de Janeiro; RN: Rio Grande do Norte; RO: Rondônia; RR: Roraima; RS: Rio Grande do Sul; SC: Santa Catarina; SE: Sergipe; SP: São Paulo; TO: Tocantins.

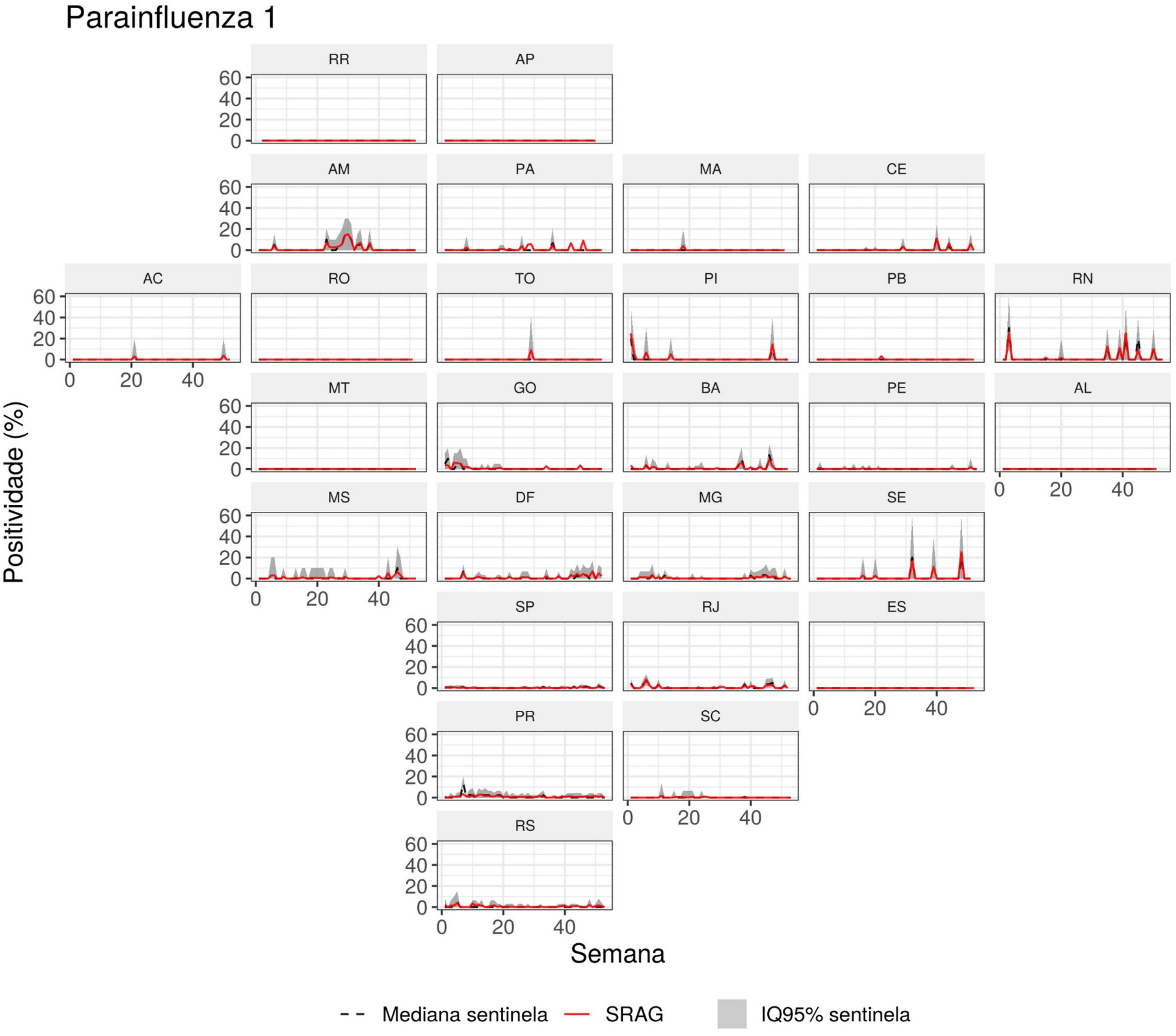

Figura S7 Positividade de parainfluenza 1 na vigilância sentinela de síndrome gripal simulada (mediana e intervalo quantílico de 95% – IQ95%) e nos casos testados de síndrome respiratória aguda grave (SRAG) por Unidade Federativa e semana epidemiológica, Brasil, 2014-2019.

Unidades Federativas: AC: Acre; AL: Alagoas; AM: Amazonas; AP: Amapá; BA: Bahia; CE: Ceará; DF: Distrito Federal; ES: Espírito Santo; GO: Goiás; MA: Maranhão; MG: Minas Gerais; MS: Mato Grosso do Sul; MT: Mato Grosso; PA: Pará; PB: Paraíba; PE: Pernambuco; PI: Piauí; PR: Paraná; RJ: Rio de Janeiro; RN: Rio Grande do Norte; RO: Rondônia; RR: Roraima; RS: Rio Grande do Sul; SC: Santa Catarina; SE: Sergipe; SP: São Paulo; TO: Tocantins.

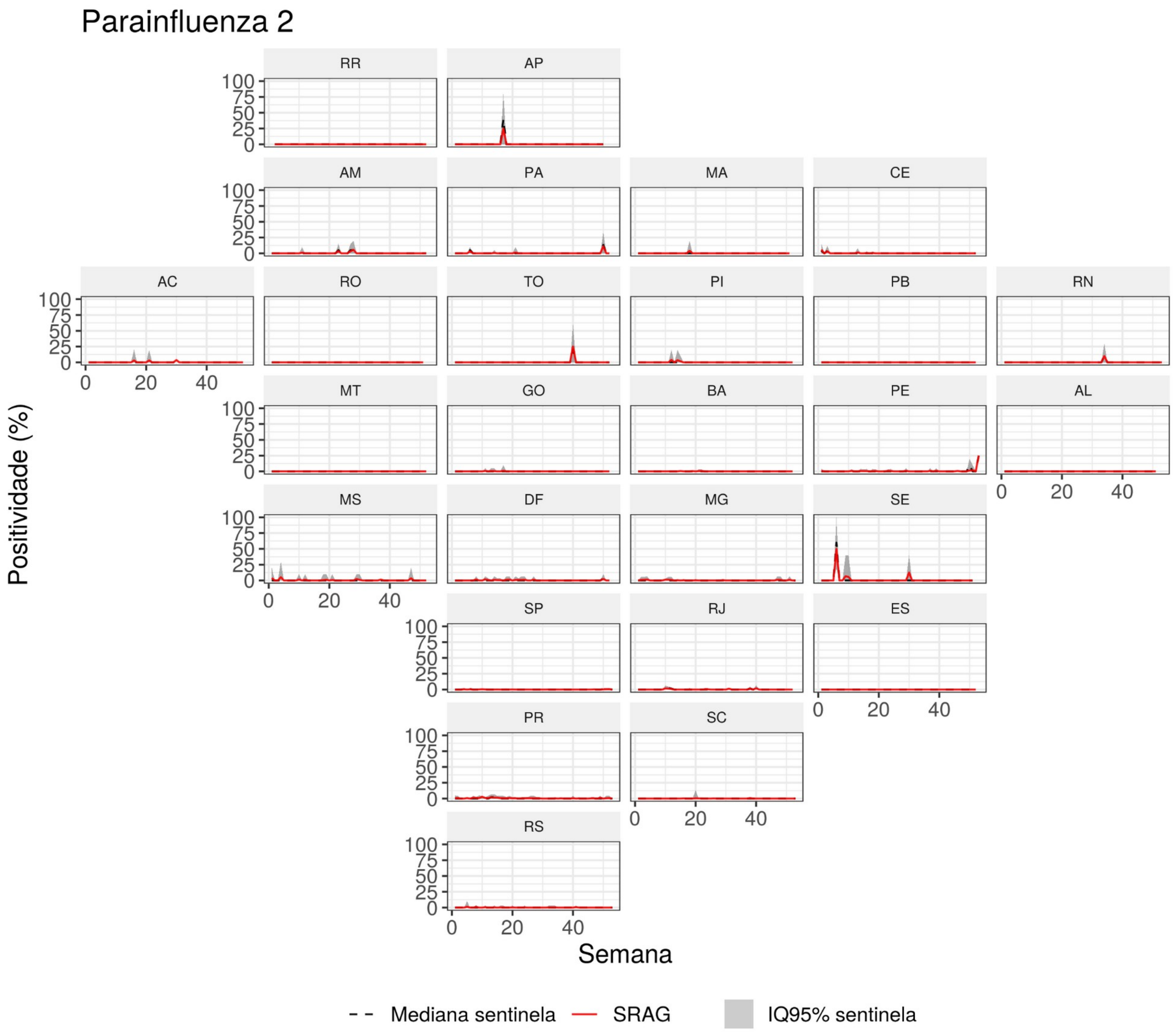

Figura S8 Positividade de parainfluenza 2 na vigilância sentinela de síndrome gripal simulada (mediana e intervalo quantílico de 95% – IQ95%) e nos casos testados de síndrome respiratória aguda grave (SRAG) por Unidade Federativa e semana epidemiológica, Brasil, 2014-2019.

Unidades Federativas: AC: Acre; AL: Alagoas; AM: Amazonas; AP: Amapá; BA: Bahia; CE: Ceará; DF: Distrito Federal; ES: Espírito Santo; GO: Goiás; MA: Maranhão; MG: Minas Gerais; MS: Mato Grosso do Sul; MT: Mato Grosso; PA: Pará; PB: Paraíba; PE: Pernambuco; PI: Piauí; PR: Paraná; RJ: Rio de Janeiro; RN: Rio Grande do Norte; RO: Rondônia; RR: Roraima; RS: Rio Grande do Sul; SC: Santa Catarina; SE: Sergipe; SP: São Paulo; TO: Tocantins.

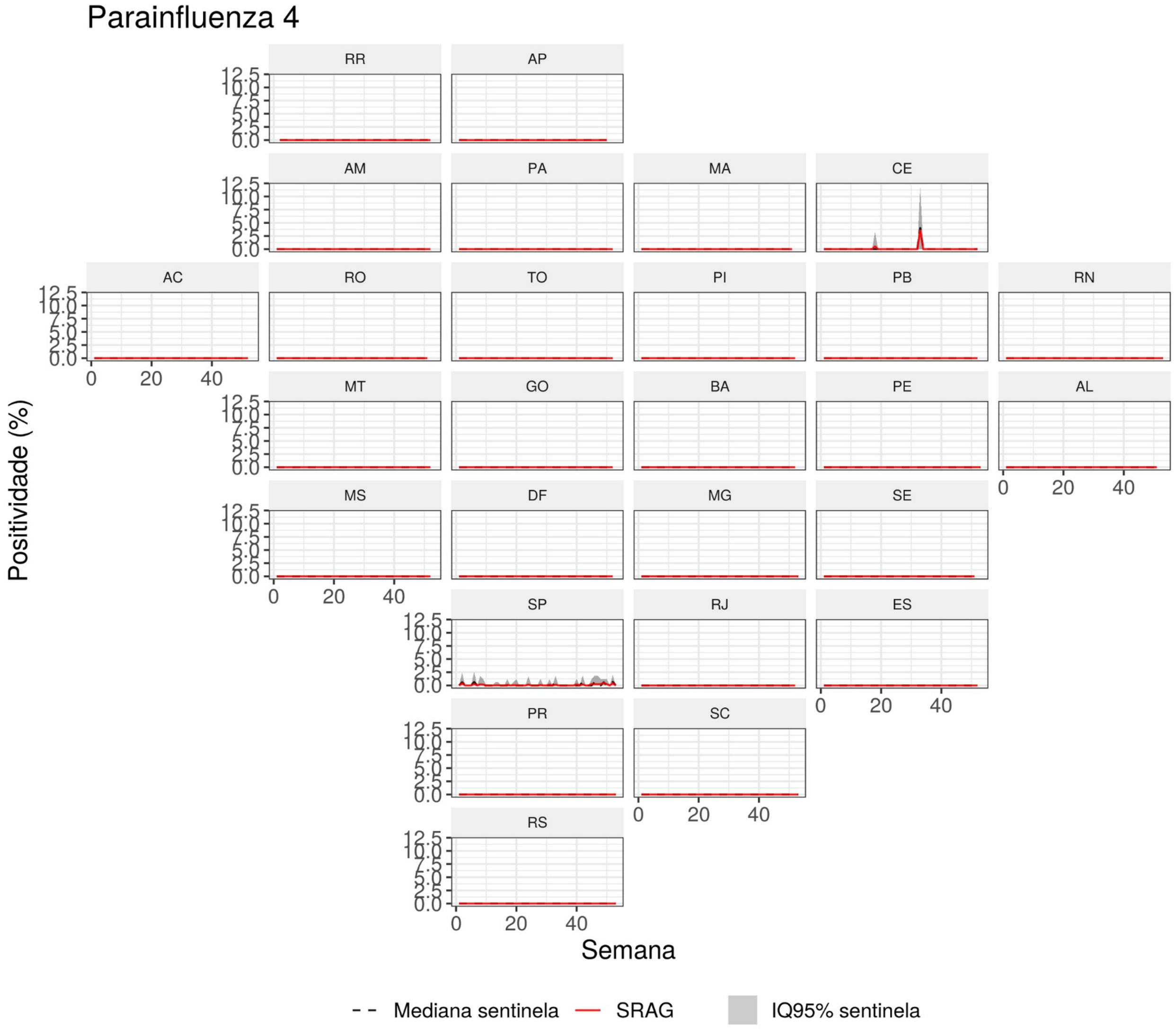

Figura S9 Positividade de parainfluenza 4 na vigilância sentinela de síndrome gripal simulada (mediana e intervalo quantílico de 95% – IQ95%) e nos casos testados de síndrome respiratória aguda grave (SRAG) por Unidade Federativa e semana epidemiológica, Brasil, 2014-2019.

Unidades Federativas: AC: Acre; AL: Alagoas; AM: Amazonas; AP: Amapá; BA: Bahia; CE: Ceará; DF: Distrito Federal; ES: Espírito Santo; GO: Goiás; MA: Maranhão; MG: Minas Gerais; MS: Mato Grosso do Sul; MT: Mato Grosso; PA: Pará; PB: Paraíba; PE: Pernambuco; PI: Piauí; PR: Paraná; RJ: Rio de Janeiro; RN: Rio Grande do Norte; RO: Rondônia; RR: Roraima; RS: Rio Grande do Sul; SC: Santa Catarina; SE: Sergipe; SP: São Paulo; TO: Tocantins.

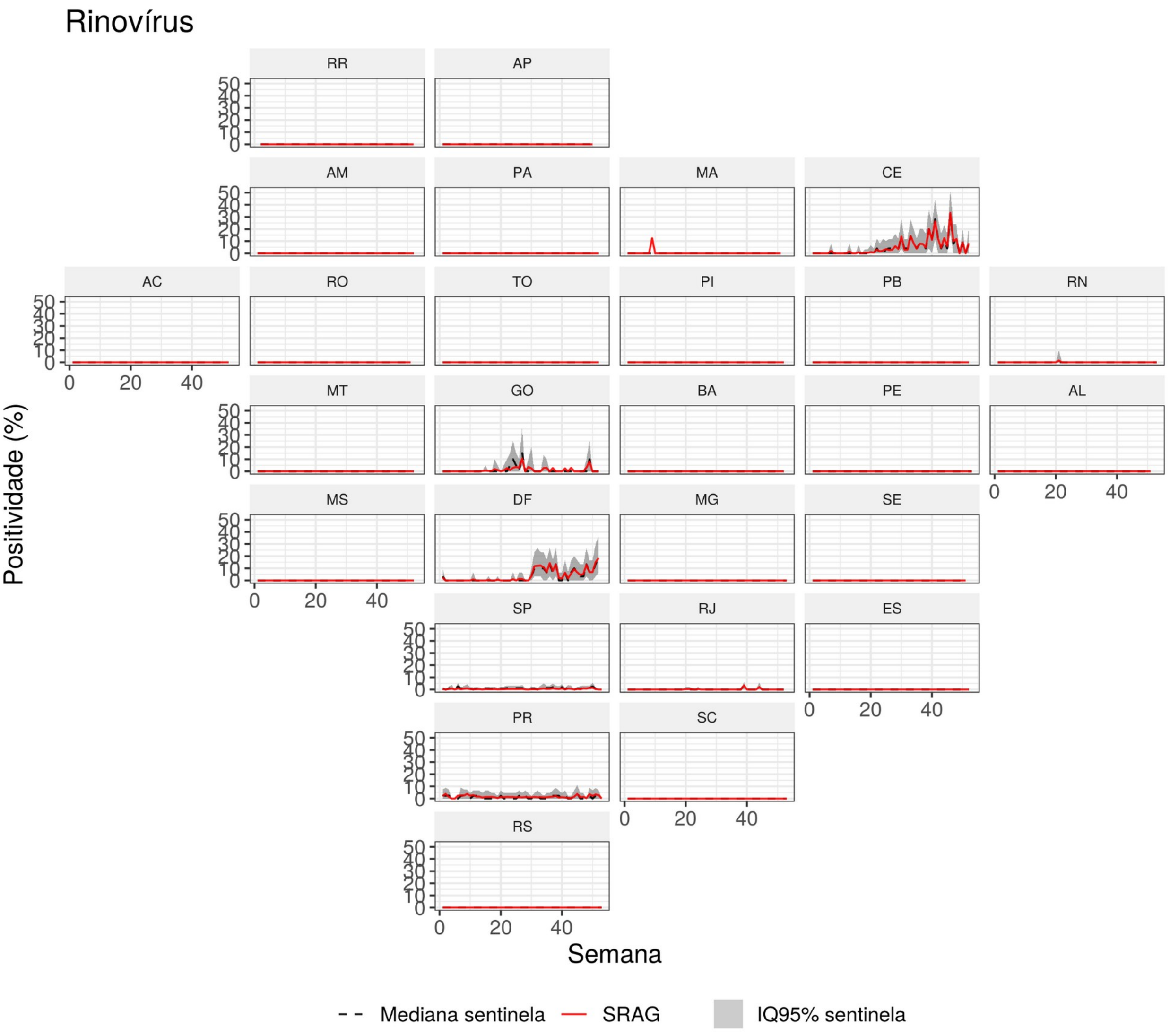

Figura S10 Positividade de rinovírus na vigilância sentinela de síndrome gripal simulada (mediana e intervalo quantílico de 95% – IQ95%) e nos casos testados de síndrome respiratória aguda grave (SRAG) por Unidade Federativa e semana epidemiológica, Brasil, 2014-2019.

Unidades Federativas: AC: Acre; AL: Alagoas; AM: Amazonas; AP: Amapá; BA: Bahia; CE: Ceará; DF: Distrito Federal; ES: Espírito Santo; GO: Goiás; MA: Maranhão; MG: Minas Gerais; MS: Mato Grosso do Sul; MT: Mato Grosso; PA: Pará; PB: Paraíba; PE: Pernambuco; PI: Piauí; PR: Paraná; RJ: Rio de Janeiro; RN: Rio Grande do Norte; RO: Rondônia; RR: Roraima; RS: Rio Grande do Sul; SC: Santa Catarina; SE: Sergipe; SP: São Paulo; TO: Tocantins.

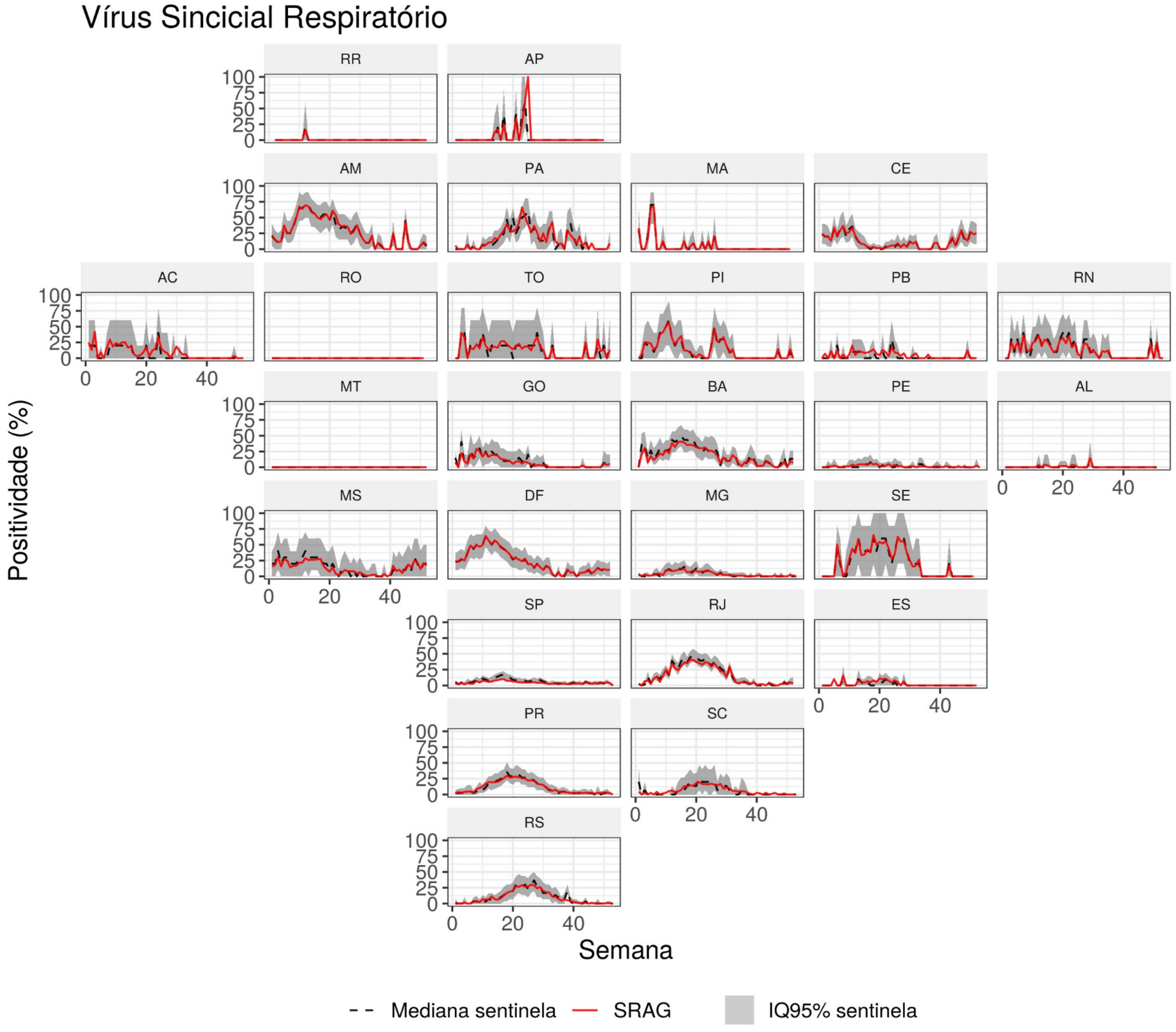

Figura S11 Positividade de vírus sincicial respiratório na vigilância sentinela de síndrome gripal simulada (mediana e intervalo quantílico de 95% – IQ95%) e nos casos testados de síndrome respiratória aguda grave (SRAG) por Unidade Federativa e semana epidemiológica, Brasil, 2014-2019.

Unidades Federativas: AC: Acre; AL: Alagoas; AM: Amazonas; AP: Amapá; BA: Bahia; CE: Ceará; DF: Distrito Federal; ES: Espírito Santo; GO: Goiás; MA: Maranhão; MG: Minas Gerais; MS: Mato Grosso do Sul; MT: Mato Grosso; PA: Pará; PB: Paraíba; PE: Pernambuco; PI: Piauí; PR: Paraná; RJ: Rio de Janeiro; RN: Rio Grande do Norte; RO: Rondônia; RR: Roraima; RS: Rio Grande do Sul; SC: Santa Catarina; SE: Sergipe; SP: São Paulo; TO: Tocantins.
