# Supplementary material for: Evaluation of the design of the influenza-like illness sentinel surveillance system in Brazil
Source: Cad Saude Publica. 2024 Jul 29;40(6):e00028823. doi: 10.1590/0102-311XEN028823 (PMC11321611; doi:10.1590/0102-311XEN028823)
Supplement: Translation

# Translation: Avaliação do desenho da vigilância sentinela de síndrome gripal no Brasil

**DOI:** 10.1590/0102-311XPT028823

**Authors:** Laís Picinini Freitas, Cláudia Torres Codeço, Leonardo Soares Bastos, Daniel Antunes Maciel Villela, Oswaldo Gonçalves Cruz, Antonio Guilherme Pacheco, Flavio Codeço Coelho, Raquel Martins Lana, Luiz Max Fagundes de Carvalho, Roberta Pereira Niquini, Walquiria Aparecida Ferreira de Almeida, Daiana Araújo da Silva, Felipe Cotrim de Carvalho, Marcelo Ferreira da Costa Gomes

**Affiliations:** 1 Programa de Computação Científica, Fundação Oswaldo Cruz, Rio de Janeiro, Brasil.; 2 Escola de Matemática Aplicada, Fundação Getulio Vargas, Rio de Janeiro, Brasil.; 3 Barcelona Supercomputing Center, Barcelona, España.; 4 Instituto Federal de Educação, Ciência e Tecnologia do Rio de Janeiro, Rio de Janeiro, Brasil.; 5 Secretaria de Vigilância em Saúde, Ministério da Saúde, Brasília, Brasil.

**Keywords:** Síndrome Respiratória Aguda Grave, Influenza Humana, Vigilância Sentinela

## Abstract

A vigilância sentinela de síndrome gripal atua no Brasil identificando os vírus respiratórios de importância para a saúde pública circulantes no país, e começou a ser implementada em 2000. Recentemente, a pandemia de COVID-19 reforçou a importância da detecção precoce de novos vírus em circulação no território brasileiro. Assim, se faz oportuna uma análise do desenho da vigilância sentinela de síndrome gripal. Para tal, simulamos uma rede sentinela, identificando os municípios que fariam parte da rede segundo os critérios definidos no desenho da vigilância sentinela de síndrome gripal, e, a partir dos dados de casos testados de síndrome respiratória aguda grave (SRAG) de 2014 a 2019, sorteamos amostras para cada município sentinela por semana epidemiológica. O sorteio foi repetido mil vezes, obtendo-se a mediana e intervalo quantílico de 95% (IQ95%) da positividade para cada vírus por Unidade Federativa e semana epidemiológica. Segundo os critérios do desenho da vigilância sentinela de síndrome gripal, unidades sentinelas estariam em 64 municípios, distribuídas principalmente em capitais e suas zonas metropolitanas, o que preconizou 690 amostras semanais. O desenho apresentou boa sensibilidade (total de 91,65%, considerando o IQ95%) para a detecção qualitativa dos vírus respiratórios, mesmo os de baixa circulação. Porém, houve importante incerteza na estimativa quantitativa de positividade, chegando a, pelo menos, 20% em 11,34% das estimativas. Os resultados aqui apresentados visam auxiliar a avaliação e a atualização do desenho da rede sentinela. Estratégias para reduzir a incerteza nas estimativas de positividade precisam ser avaliadas, assim como a necessidade de maior cobertura espacial.

## Introdução

A vigilância sentinela de síndrome gripal começou a ser implementada no Brasil em 2000, com os objetivos de identificar os vírus respiratórios de importância em saúde pública circulantes no país, guiar a composição da vacina sazonal contra a influenza e gerar alertas de eventos incomuns, como a emergência de um novo vírus ^1^ ^,^ ^2^ ^,^ ^3^. Ela integra a vigilância nacional da influenza e de outros vírus respiratórios, da qual também faz parte a vigilância universal de casos hospitalizados e óbitos por síndrome respiratória aguda grave (SRAG), implementada em 2009. Os dados de ambas as vigilâncias são captados em um sistema de informação oficial denominado Sistema de Vigilância Epidemiológica da Gripe (SIVEP-Gripe), que integra o Departamento de Informática do Sistema Único de Saúde (DATASUS) do Ministério da Saúde.

Nele, o serviço da vigilância sentinela de síndrome gripal é formado por uma rede de unidades de saúde, seguindo as orientações da Organização Mundial da Saúde (OMS) ^1^ ^,^ ^4^. Existem outras formas de vigilância sentinela, como em países onde ela é composta por profissionais de saúde ^5^. De acordo com dados do Centro Europeu de Prevenção e Controle de Doenças (European Centre for Disease Prevention and Control - ECDC), desde 2015 todos os estados membros reportam dados para a vigilância de influenza sazonal a partir de casos de síndrome gripal na atenção primária ^5^. Diversos deles utilizam não apenas unidades sentinela, mas também dados de reporte voluntário das unidades não sentinela para o monitoramento de situação e a identificação viral, cenário que também se observa em países que compõem a região europeia da OMS ^6^. Nos Estados Unidos, o Centro de Controle e Prevenção de Doenças (Centers for Disease Control and Prevention - CDC) também se vale de vigilância sentinela de síndrome gripal para monitoramento de casos de infecção respiratória que requerem atendimento ambulatorial ^7^. O CDC destaca que apenas um subconjunto de casos recolhe amostra para identificação viral, sendo o foco principal da vigilância sentinela o monitoramento da tendência e do volume de casos de infecções respiratórias em geral, não sendo vírus específica ^7^. Na Europa também se adota estratégia amostral para coleta de amostra para testagem, não sendo testados todos casos de síndrome gripal notificados na rede sentinela ^8^. Nas Américas, dos 38 países e territórios avaliados até 2021 pela Organização Pan-Americana da Saúde (OPAS), 25 possuíam vigilância de síndrome gripal e 31 de SRAG, sendo que a ampla maioria utiliza a estratégia sentinela e testagem laboratorial de apenas um subconjunto dos casos de síndrome gripal ^9^.

No âmbito da necessidade de fortalecimento da vigilância sentinela no Brasil, após a pandemia de influenza A(H1N1)pdm09 foi publicada a *Portaria nº 183* do Ministério da Saúde ^10^, de 30 de janeiro de 2014, a qual determina, no capítulo V, art. 28, § 1º, os critérios para a distribuição das unidades de saúde que formam a rede de vigilância sentinela de síndrome gripal. Essas unidades são obrigatoriamente serviços de saúde, que devem incluir unidades de urgência e emergência e atender pessoas de todas as faixas etárias. Até o ano de 2019, a vigilância sentinela definia um caso de síndrome gripal como um indivíduo com febre, mesmo que referida, acompanhada de tosse ou dor de garganta e com início dos sintomas nos últimos sete dias, atendido em uma unidade de saúde sentinela. Quanto à análise laboratorial, eram preconizadas cinco amostras de secreção nasofaríngea de casos de síndrome gripal por semana em cada unidade sentinela ^2^ ^,^ ^10^. Essas amostras são enviadas aos laboratórios públicos para serem testadas contra um painel de vírus respiratórios que, com a inclusão de novos vírus, se modificou ao longo dos anos. Com essa amostragem, espera-se que os dados captados sejam representativos da Unidade Federativa.

Diferentemente da síndrome gripal, que tem uma vigilância por amostragem, todos os casos e óbitos que atendem a definição de SRAG devem ser notificados no SIVEP-Gripe, além de serem testados para o painel de vírus respiratórios independentemente da unidade de saúde. Até 2019, casos de SRAG eram definidos como casos hospitalizados ou que foram a óbito, independentemente de hospitalização prévia, com os mesmos sintomas da síndrome gripal acrescidos de dispneia ou saturação de O_2_ menor que 95% ou desconforto respiratório ^3^. Os vírus respiratórios encontrados na vigilância de SRAG não necessariamente representam a população viral circulante, uma vez que alguns levam a quadros clínicos mais leves que não seriam facilmente identificados pela vigilância de SRAG. Dessa forma, a vigilância sentinela de síndrome gripal (casos leves) e a vigilância universal de SRAG (casos graves) são complementares na medida em que cobrem um espectro amplo de síndromes respiratórias.

A pandemia de COVID-19, doença causada pelo SARS-CoV-2, reforçou ainda mais a importância da vigilância de síndromes respiratórias para detecção precoce de circulação de novos vírus no território brasileiro. O vírus emergente se disseminou pelo país muito rapidamente, alcançando regiões distantes dos grandes centros urbanos em poucas semanas ^11^. Mediante as mudanças nos padrões de circulação humana e o aumento do risco de introdução e espalhamento de novos vírus e variantes, faz-se oportuna uma análise da estrutura da rede sentinela atualmente proposta, a fim de identificar fortalezas e fragilidades que possam subsidiar novos desenhos. Portanto, o objetivo principal deste trabalho é avaliar o desempenho do desenho da vigilância sentinela de síndrome gripal quanto à sua capacidade de detecção da prevalência de vírus respiratórios por Unidade Federativa.

## Metodologia

### Dados

No Brasil, a fonte de dados disponível com melhor cobertura de informação sobre a circulação de vírus respiratórios provém da vigilância universal de SRAG. Essa é capaz de monitorar a sazonalidade das síndromes gripais no país ^12^ e se tornou imprescindível para monitorar a expansão do SARS-CoV-2 ^13^ ^,^ ^14^ ^,^ ^15^ e auxiliar no planejamento da vacinação nacional contra a COVID-19 ^16^. Na ausência de fonte não enviesada, assumimos neste trabalho que a distribuição de casos de SRAG por subtipo viral captado pela vigilância universal é representativa da distribuição espacial verdadeira de vírus respiratórios no país. Assim, espera-se que uma rede sentinela com distribuição adequada seja capaz de detectar essa distribuição viral por Unidade Federativa, que é a resolução espacial para a qual ela foi desenhada para ser representativa ^2^ ^,^ ^3^ ^,^ ^17^ ^,^ ^18^. Para testar essa hipótese, nós: (1) identificamos quais seriam os municípios elegíveis para compor a rede sentinela; (2) calculamos quantas unidades sentinelas e quantas amostras semanais seriam preconizadas em cada município elegível; e (3) simulamos o processo de coleta de dados feito pela rede sentinela num cenário em que a população viral, por semana e por Unidade Federativa, provém de uma amostra com reposição da composição viral dos casos de SRAG na mesma data e local. Para tal, tomamos como base a *Portaria nº 183* do Ministério da Saúde ^10^, capítulo V, art. 28, §1º, e outros documentos do Ministério da Saúde que complementam as informações dela ^2^ ^,^ ^18^.

Os dados de casos de SRAG registrados no SIVEP-Gripe foram obtidos no âmbito do InfoGripe (http://infogripe.fiocruz.br/), que é uma iniciativa para monitorar e apresentar níveis de alerta para os casos de SRAG ^19^. O projeto é fruto de uma parceria entre pesquisadores do Programa de Computação Científica, Fundação Oswaldo Cruz (PROCC/FIOCRUZ), da Escola de Matemática Aplicada, Fundação Getulio Vargas (EMap/FGV), e da Secretaria de Vigilância em Saúde, Ministério da Saúde.

Foram utilizados os dados dos casos de SRAG de 2014 a 2019 de acordo com o ano de início dos sintomas, totalizando 214.162 registros. Destes, foram selecionados apenas os casos que foram testados laboratorialmente, resultando em 178.106 casos (83,2% do total). No período estudado, os testes laboratoriais disponíveis cobriam adenovírus, influenza A, influenza B, parainfluenza 1, parainfluenza 2, parainfluenza 3, parainfluenza 4, vírus sincicial respiratório, metapneumovírus, rinovírus e bocavírus. A positividade de cada um entre os casos testados de SRAG, de 2014 a 2019, está apresentada no Material Suplementar (Figura S1 https://cadernos.ensp.fiocruz.br/static//arquivo/csp-0288-23-sup-ept028823_4456.pdf).

Também foram utilizadas as estimativas populacionais para o ano de 2019 do Ministério da Saúde, disponibilizadas pelo DATASUS (https://datasus.saude.gov.br/).

### Análises

#### Identificação dos municípios elegíveis para compor a rede sentinela

Inicialmente, foram identificados quais municípios seriam elegíveis para compor a rede da vigilância sentinela de síndrome gripal ^10^, que são: (1) todas as capitais das Unidades Federativas; (2) municípios com população maior que 300 mil habitantes na Região Sul; e (3) municípios com mais de 300 mil habitantes nas regiões metropolitanas das capitais das demais regiões. Em seguida, calculou-se o número de unidades sentinelas e amostras semanais preconizadas para cada município ^2^ ^,^ ^10^, com cinco amostras semanais para cada unidade sentinela, sendo: (1) uma unidade a cada 500 mil habitantes nas capitais; e (2) uma unidade nos demais municípios da rede. Também se calculou o número de amostras semanais esperadas a cada 1 milhão de habitantes por Unidade Federativa.

#### Vigilância sentinela simulada

O conjunto de 178.106 casos de SRAG testados laboratorialmente e notificados no SIVEP-Gripe foi estratificado por semana e município de notificação (período de 2014 a 2019). A partir desse conjunto, foram sorteados *n*_*m,t*_ casos em cada município sentinela *m* e semana epidemiológica *t*, seguindo os critérios da estratégia da vigilância sentinela descritos acima. No sorteio, assume-se que todos os casos notificados no município sentinela têm a mesma probabilidade de serem captados pelas unidades sentinelas presentes nele. Por outro lado, casos notificados em municípios sem unidades sentinelas têm probabilidade zero de serem captados pela rede sentinela. Para obter medidas de incerteza, o processo de sorteio (com reposição) foi repetido mil vezes. Ao conjunto de dados resultante, chamamos de vigilância sentinela simulada.

A partir do total de casos de SRAG capturados pela sentinela simulada, calculou-se *P*_*v,t,i,k*_ , definido como a positividade do vírus *v* na semana *t*, para cada Unidade Federativa *i* e repetição *k* (Equação 1). Como o processo foi repetido mil vezes, sendo *k* = 1,2, …, 1.000, tem-se mil valores que descrevem a distribuição de positividade de cada vírus por Unidade Federativa e semana. A partir de *P*_*v,t,i,k*_ , calculou-se a mediana e o intervalo quantílico de 95% (IQ95%) da positividade para cada vírus *v* por semana *t* e Unidade Federativa *i*.

$${Ρ}_{v,t,i,k}=\frac{\sum_{m\in i}{positivos}_{m,v,t,k}}{\sum_{m\in i}n_{m,t}}\times 100$$

Para analisar a qualidade do indicador gerado pela sentinela simulada, foi calculado o erro absoluto *E*_*v,t,i,k*_ (Equação 2) comparando cada valor da positividade *P*_*v,t,i,k*_ da sentinela simulada com a positividade “verdadeira” (*ϕ*_*v,i,t*_ ), calculada a partir dos dados totais de SRAG presentes no universo de casos notificados e testados laboratorialmente.

$$E_{v,t,i,k}=\left|P_{v,t,i,k}-\phi_{v,i,t}\right|$$

A partir de *E*_*v,t,i,k*_ , calculou-se o erro absoluto mediano para cada vírus *v* por semana *t* e Unidade Federativa *i*.

Foram confeccionados mapas para comparar os erros nas estimativas de positividade entre Unidade Federativa e entre períodos do ano de maior ou menor atividade de vírus respiratórios. Utilizando o *Moving Epidemic Method* (MEM) ^20^ ^,^ ^21^, implementado no InfoGripe ^22^, períodos de cada ano de cada Unidade Federativa foram classificados como epidêmicos (semanas de maior atividade) ou interepidêmicos (semanas de menor atividade). Esse método de classificação da atividade de influenza também é utilizado na rotina do ECDC ^20^. Para comparar a performance da sentinela nos períodos de alta e baixa atividade, utilizou-se a média dos erros absolutos para cada vírus por período, calculada como a soma dos erros absolutos dividida pelo número de semanas.

Como são mais de dez vírus respiratórios testados laboratorialmente pela vigilância, classificamos os vírus em dois grupos (de maior e de menor circulação) para facilitar a visualização dos resultados, e selecionamos um de cada para apresentação. Para essa classificação, consideramos o ponto de corte de 2% na positividade no universo de dados de SRAG (Material Suplementar - Figura S1 https://cadernos.ensp.fiocruz.br/static//arquivo/csp-0288-23-sup-ept028823_4456.pdf). Os dois vírus selecionados para representar os grupos de maior e de menor circulação, respectivamente, foram influenza A (positividade = 16,4%) e parainfluenza 3 (positividade = 1,2%). Os resultados referentes aos demais vírus estão disponíveis no Material Suplementar (Figuras S2-S11; https://cadernos.ensp.fiocruz.br/static//arquivo/csp-0288-23-sup-ept028823_4456.pdf).

Para organizar e analisar os dados, utilizamos o R versão 4.0.4 (http://www.r-project.org) e o pacote *tidyverse* ^23^. Os gráficos e mapas foram confeccionados no R, utilizando o pacote *ggplot2* ^24^.

### Aspectos éticos

Este trabalho utilizou dados não identificáveis que podem ser encontrados de forma irrestrita e pública na página do OpenDATASUS (https://opendatasus.saude.gov.br/).

## Resultados

De acordo com o desenho da vigilância sentinela de síndrome gripal, a estratégia incluiria 138 unidades em 64 municípios, preconizando um total de 690 amostras por semana. Desses 64, dez estariam concentrados apenas na região metropolitana de São Paulo. Em média, eles deveriam ter duas unidades sentinelas, variando de 1 a 25. A lista de municípios com o número preconizado de unidades e de amostras, de acordo com o desenho, está no Material Suplementar (Tabela S1 https://cadernos.ensp.fiocruz.br/static//arquivo/csp-0288-23-sup-ept028823_4456.pdf).

Este desenho prevê um maior número de amostras semanais coletadas nas Unidades Federativas das regiões Sul e Sudeste, nas quais se concentra a maior parte da população brasileira (Figura 1a). A amostragem proposta corresponde a 3,3 amostras por milhão de habitantes, variando de 1,4 a 9,9 por Unidade Federativa (Figura 1b). As com menor número de amostras por população seriam o Maranhão (1,4 amostra por milhão de habitantes), Mato Grosso (1,4 amostra por milhão de habitantes) e Minas Gerais (1,8 amostra por milhão de habitantes), enquanto que aquelas com maior número seriam o Distrito Federal (9,9 amostras por milhão de habitantes), Roraima (8,2 amostras por milhão de habitantes) e Amapá (5,9 amostras por milhão de habitantes). Na Figura 1b também estão destacados os municípios que atendem os critérios para integrarem a rede sentinela, conforme foi desenhado. Na maioria das Unidades Federativas, esses municípios correspondem às capitais ou aos vizinhos às capitais. Apenas no Paraná se preconizam municípios sentinelas mais distribuídos pelo território.

**Figura 1:** Amostras semanais preconizadas pelo desenho da vigilância sentinela de síndrome gripal, por Unidade Federativa, e por 1 milhão de habitantes, Brasil.

CO: Centro-oeste; N: Norte; NE: Nordeste; S: Sul; SE: Sudeste.

A distribuição dos erros absolutos do índice de positividade da vigilância sentinela simulada por Unidade Federativa e semana para todos os vírus, e especificamente para influenza A e para parainfluenza 3, está na Figura 2. Observa-se que nas Unidades Federativas das regiões Sul e Sudeste (excetuando-se o Espírito Santo) e no Distrito Federal, a sentinela simulada apresentou erros absolutos pequenos, com distribuição mais concentrada em valores próximos de zero. Apenas no Amapá o limite superior da distribuição dos erros absolutos excedeu 50%. No Mato Grosso e em Roraima, os erros apresentaram uma faixa mais larga de distribuição.

**Figura 2:** Distribuição dos erros absolutos da positividade viral detectada pela vigilância sentinela simulada em relação aos valores reais obtidos a partir do universo da vigilância de síndrome respiratória aguda grave (SRAG), para todos os vírus (Total), para influenza A (Flu A) e para parainfluenza 3 (Para 3), por semana epidemiológica e Unidade Federativa, Brasil, 2014-2019.

Unidades Federativas: AC: Acre; AL: Alagoas; AM: Amazonas; AP: Amapá; BA: Bahia; CE: Ceará; DF: Distrito Federal; ES: Espírito Santo; GO: Goiás; MA: Maranhão; MG: Minas Gerais; MS: Mato Grosso do Sul; MT: Mato Grosso; PA: Pará; PB: Paraíba; PE: Pernambuco; PI: Piauí; PR: Paraná; RJ: Rio de Janeiro; RN: Rio Grande do Norte; RO: Rondônia; RR: Roraima; RS: Rio Grande do Sul; SC: Santa Catarina; SE: Sergipe; SP: São Paulo; TO: Tocantins.

Observa-se na Figura 3 que, de forma geral, os erros absolutos foram maiores no período interepidêmico e para o vírus influenza A (de maior circulação), quando em comparação com o parainfluenza 3 (de menor circulação). Para o influenza A, os erros absolutos variaram de 3,6% (Maranhão) a 29,9% (Mato Grosso) no período interepidêmico, e de 1,7% (Roraima) a 14,7% (Sergipe) no período epidêmico. Já para o parainfluenza 3, os erros absolutos variaram de 0,4% (Acre) a 16,9% (Sergipe) no período interepidêmico, e de 0,1% (Minas Gerais) a 2,9% (Amapá) no período epidêmico. O vírus parainfluenza 3 não foi detectado nos dados totais de SRAG em oito Unidades Federativas no período interepidêmico, e em nove no período epidêmico (mostradas em cinza nas Figura 3b e 3d).

**Figura 3:** Erros absolutos da vigilância sentinela de síndrome gripal simulada em relação aos dados totais de síndrome respiratória aguda grave, para influenza A e parainfluenza 3, por período epidemiológico e Unidade Federativa, Brasil, 2014-2019.

A presença de vírus respiratórios (ou seja, positividade maior que zero) foi corretamente detectada pela vigilância sentinela simulada em 91,65% do total de observações, considerando o IQ95%. Levando em conta apenas a mediana da positividade da vigilância sentinela, esse valor cai para 57,97%. Em geral, os valores verdadeiros da positividade para influenza A e parainfluenza 3 estiveram dentro do IQ95% da positividade da vigilância sentinela simulada (Figura 4). Para o vírus influenza A, São Paulo, Rio de Janeiro, Paraná e Rio Grande do Sul apresentaram menores incertezas, enquanto em Unidades Federativas como Amapá, Rondônia, Mato Grosso e Sergipe a faixa do IQ95% foi maior que 50% em algumas semanas (Figura 4a). Em sete Unidades Federativas (Roraima, Maranhão, Rondônia, Paraíba, Mato Grosso, Alagoas e Espírito Santo), o vírus parainfluenza 3 não foi detectado no universo de dados de SRAG em nenhuma semana. Apesar da baixa positividade, a vigilância sentinela simulada foi capaz de detectar a presença do parainfluenza 3 quando ele circulava (Figura 4b).

**Figura 4:** Positividade de influenza A e parainfluenza 3 obtida pela vigilância sentinela de síndrome gripal simulada (mediana e intervalo quantílico de 95% - IQ95%), em comparação com a positividade de referência obtida a partir do universo de casos notificados de síndrome respiratória aguda grave (SRAG) por Unidade Federativa e semana epidemiológica, Brasil, 2014-2019.

Unidades Federativas: AC: Acre; AL: Alagoas; AM: Amazonas; AP: Amapá; BA: Bahia; CE: Ceará; DF: Distrito Federal; ES: Espírito Santo; GO: Goiás; MA: Maranhão; MG: Minas Gerais; MS: Mato Grosso do Sul; MT: Mato Grosso; PA: Pará; PB: Paraíba; PE: Pernambuco; PI: Piauí; PR: Paraná; RJ: Rio de Janeiro; RN: Rio Grande do Norte; RO: Rondônia; RR: Roraima; RS: Rio Grande do Sul; SC: Santa Catarina; SE: Sergipe; SP: São Paulo; TO: Tocantins.

## Discussão

Neste trabalho, buscamos avaliar se, no Brasil, a vigilância sentinela de síndrome gripal como foi desenhada ^2^ ^,^ ^10^ seria capaz de identificar a prevalência de diferentes vírus respiratórios por Unidade Federativa e semana. Consideramos os dados da vigilância de SRAG, que tem cobertura em todo o país, como representante da prevalência “real” dos vírus respiratórios e, a partir desses dados, simulamos a captação de dados pela vigilância sentinela tal qual está proposta na referida portaria. De forma geral, encontramos que a vigilância sentinela simulada detectou qualitativamente a presença de vírus respiratórios nas Unidades Federativas, porém as estimativas de positividade não foram precisas.

A avaliação aqui apresentada considera uma implementação perfeita da rede sentinela preconizada tal qual foi desenhada ^2^ ^,^ ^10^. Portanto, não é uma avaliação de como ela realmente está em funcionamento. Na prática, há desafios para manter as unidades sentinelas ativas e o envio de cinco amostras semanais preconizadas, assim como problemas provenientes de operacionalização como qualidade da amostra nasofaríngea coletada, seleção dos casos de síndrome gripal, armazenamento e transporte das amostras, acesso aos laboratórios, qualidade dos dados registrados, entre outros ^25^ ^,^ ^26^. Além disso, consideramos o cumprimento de 100% da meta de cinco amostras semanais, enquanto um mínimo de 80% é exigido para o repasse de fundos. Isso implica que os resultados aqui apresentados correspondem a um limite superior da performance desse sistema.

A partir de simulações realizadas, verificou-se a capacidade da rede sentinela preconizada para monitoramento temporal e espacial da composição da população viral a nível de Unidade Federativa, isso é, com a detecção dos tipos virais presentes. A boa sensibilidade apresentada para o parainfluenza 3 indica que a vigilância sentinela é adequada para detectar vírus menos prevalentes ou de ocorrência intermitente. Esse resultado é importante para cumprir o objetivo de caracterização dos vírus visando composição de vacinas, por exemplo.

Em geral, observamos menores erros absolutos em Unidades Federativas das regiões Sul e Sudeste e do Distrito Federal (Figura 2). Isso provavelmente reflete a representatividade da amostragem da rede sentinela, uma vez que nesses locais são preconizadas mais amostras semanais (Figura 1a) e/ou há uma elevada quantidade de amostras por 1 milhão de habitantes (Figura 1b). Em relação à estimativa de positividade pela sentinela simulada, observa-se uma larga incerteza para a maioria dos vírus na maior parte das Unidades Federativas (Figura 4). É importante lembrar que as estimativas aqui calculadas provêm de uma aplicação ideal do modelo preconizado, sem perdas de amostras ou unidades. A incerteza das estimativas de positividade foram altas em muitas semanas para algumas Unidades Federativas. Além disso, quando a positividade nos dados de SRAG é zero, não é possível avaliar a precisão das estimativas. De forma geral, esses resultados sugerem que a rede sentinela preconizada é inadequada para a caracterização quantitativa da prevalência. Uma possível explicação para isso decorre do viés causado pela não inclusão de outros municípios na rede sentinela. Por exemplo, 86,5% dos casos de SRAG estão em municípios não cobertos pela rede sentinela.

De acordo com o SIVEP-Gripe, em 2017, havia 115 unidades sentinelas no país (variando de uma a sete unidades por município), 16,6% a menos do que seria esperado de acordo com o desenho ^2^ ^,^ ^10^. Além disso, elas estavam distribuídas em 67 municípios, dos quais apenas 42 (64,6%) seriam selecionados se obedecidos os critérios. Observa-se que em sua presente formação, há uma concentração de unidades sentinelas em algumas Unidades Federativas (São Paulo, Rio de Janeiro, Paraná e Rio Grande do Sul), enquanto há grandes espaços descobertos no país. De acordo com o desenho da vigilância sentinela de síndrome gripal, apenas na Região Sul há previsão de estabelecimento de unidades sentinelas no interior dos estados ^2^ ^,^ ^10^. Nas demais, somente as capitais e alguns municípios das regiões metropolitanas são contemplados. Ainda assim, mesmo nos estados do Sul, percebe-se que só no Paraná haveria municípios elegíveis com maior dispersão territorial, contemplando as regiões leste, oeste e norte do estado. Em Santa Catarina e no Rio Grande do Sul, os municípios elegíveis fora da região metropolitana das capitais se concentram no litoral, além da região serrana no Rio Grande do Sul. Toda a região central e oeste desses dois estados fica descoberta. Entre as 118 macrorregiões de saúde do país, 80 (67,8%) não teriam nenhuma representação na rede sentinela segundo os critérios preconizados.

Em caso de revisão do protocolo atual de distribuição de unidades sentinelas no Brasil, sugerimos fazer uso de simulações para confrontar diferentes protocolos e para avaliação de seu custo-efetividade e eficácia. Existem propostas na literatura que utilizam redes de mobilidade para identificar pontos estratégicos ^27^. Outro caminho de desenvolvimento é a utilização de ponderação para corrigir estimativas de positividade ^28^ ^,^ ^29^. Também podem ser explorados modelos alternativos de redes sentinelas, que combinam uma representatividade populacional com uma cobertura geográfica mais uniforme ^30^.

A dinâmica espaço-temporal de vírus respiratórios é complexa e variável, com forte influência do clima, das características populacionais e do padrão de mobilidade das populações ^12^ ^,^ ^31^. Além disso, parâmetros globais de emergência e circulação viral também determinam fortemente a dinâmica epidemiológica nacional. A emergência da COVID-19 mostrou a importância das redes sentinelas para o monitoramento a longo prazo da caracterização virológica do SARS-CoV-2, assim como é feito com a influenza.
